# Translation efficiency is a determinant of the magnitude of miRNA-mediated repression

**DOI:** 10.1038/s41598-017-13851-w

**Published:** 2017-11-02

**Authors:** Kyle A. Cottrell, Pawel Szczesny, Sergej Djuranovic

**Affiliations:** 1 Department of Cell Biology and Physiology, Washington University School of Medicine, 600 South Euclid Avenue, Campus Box 8228, St. Louis, MO 63110 USA; 2 Institute of Biochemistry and Biophysics, Polish Academy of Sciences, Pawińskiego 5a, 02-106 Warsaw, Poland; 3 Faculty of Biology, University of Warsaw, Miecznikowa 1, 02-096 Warsaw, Poland

## Abstract

MicroRNAs are well known regulators of mRNA stability and translation. However, the magnitude of both translational repression and mRNA decay induced by miRNA binding varies greatly between miRNA targets. This can be the result of *cis* and *trans* factors that affect miRNA binding or action. We set out to address this issue by studying how various mRNA characteristics affect miRNA-mediated repression. Using a dual luciferase reporter system, we systematically analyzed the ability of selected mRNA elements to modulate miRNA-mediated repression. We found that changing the 3′UTR of a miRNA-targeted reporter modulates translational repression by affecting the translation efficiency. This 3′UTR dependent modulation can be further altered by changing the codon-optimality or 5′UTR of the luciferase reporter. We observed maximal repression with intermediate codon optimality and weak repression with very high or low codon optimality. Analysis of ribosome profiling and RNA-seq data for endogenous miRNA targets revealed translation efficiency as a key determinant of the magnitude of miRNA-mediated translational repression. Messages with high translation efficiency were more robustly repressed. Together our results reveal modulation of miRNA-mediated repression by characteristics and features of the 5′UTR, CDS and 3′UTR.

## Introduction

MicroRNAs are short, endogenous non-coding RNAs that along with associated Argonaute proteins form the miRNA-induced silencing complex (miRISC) which acts by inhibiting translation and causing mRNA decay^1–4^. The magnitude of translational repression and mRNA decay for each miRNA target can vary greatly^5–11^. The variation in repression for some targets can be explained by poor miRNA binding^12^, or RNA binding proteins (RBPs) modulating repression^13–15^. It is well appreciated that alternative transcription start site selection, splicing and polyadenylation can lead to transcript variants that differ by their 5′untranslated region (UTR), coding sequence (CDS) and/or 3′UTR^16–18^. In some cases, these transcript isoforms have altered repression by miRNAs^9,19^. We hypothesized that mRNA elements such as the 5′UTR, CDS and 3′UTR could modulate miRNA-mediated repression; to address this we systematically analyzed the effects of various mRNA elements on the magnitude of miRNA-mediated repression.

The 5′UTR, CDS and 3′UTR are important regulatory regions of the mRNA. Structure within the 5′UTR has been shown to affect mRNA translation by impeding the initiation process^20,21^. The presence of upstream translation start sites and upstream open reading frames (uORF) has been shown to repress translation^22–26^. Along with these repressive elements the 5′UTR is also home to binding sites for RBPs that can act on mRNA translation and stability^27^. Like the 5′UTR the 3′UTR is an important regulatory region. The 3′UTR typically contains binding sites for many RBPs and miRNAs. The RBPs that bind to the 3′UTR can influence the translation, stability and localization of the mRNA^28,29^. Sandwiched between the 5′ and 3′UTR is the CDS. The CDS is a series of mRNA codons that are translated into a protein product. In recent years, it has become apparent that the stability and translation of many mRNAs is regulated by their unique codon usage^30–34^. Messages with more optimal codons have a faster translation elongation rate and tend to be more stable^30–34^. Together, the UTRs and the CDS regulate the stability and translation of the mRNA.

Our systematic analysis showed that the magnitude of miRNA-mediated repression is dependent on the translational efficiency of the non-targeted reporter; a characteristic which can be modulated by changing the 3′UTR, codon optimality of the CDS, and 5′ UTR. Additional analysis of whole genome mRNA-seq and ribosome profiling data revealed that translation efficiency of the target mRNA is also a determinant of the magnitude of miRNA-mediated repression. Our data indicate that variation in the magnitude of miRNA-mediated translational repression observed in previous reporter and global studies^6,8,11,35,36^ can be, in part, explained by the variation in translation efficiency of the targeted message or influenced by the composition of the 5′UTR, CDS and 3′UTR.

## Results

### The 3′UTR modulates translatability and miRNA-mediated repression

**Figure 1:**
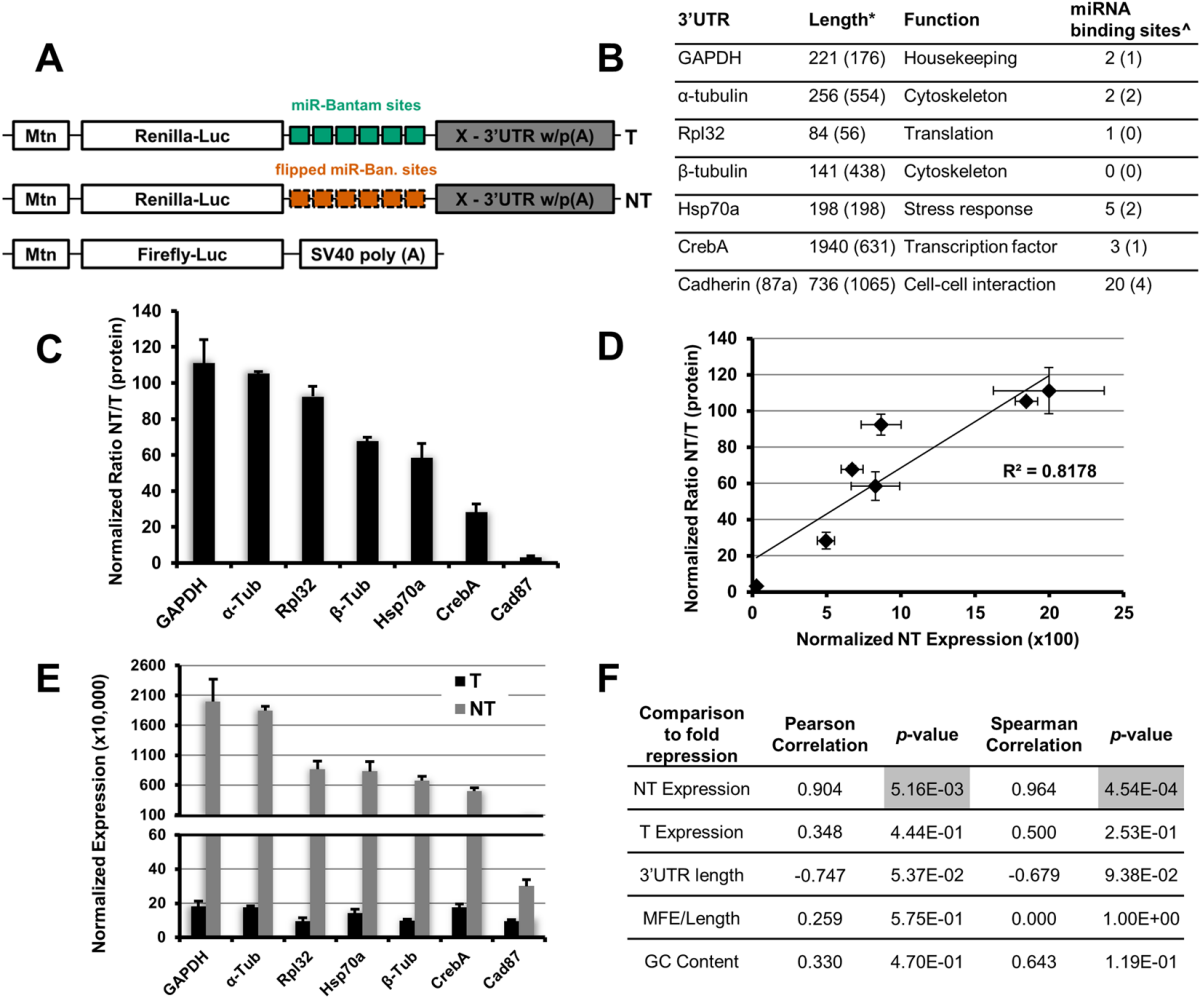
3′UTR influences translatability and miRNA-mediated repression. (**A**) Schematic describing the reporters used for this study. The 3′UTR of each of the genes in (**B**) were cloned downstream of the miRNA target sites yielding a T and NT reporter for each 3′UTR. Mtn designates the metallothionein promoter. (**B**) Table describes some attributes of the 3′UTRs used in this study. *The length of the 3′UTR as reported by Flybase (http://flybase.org/)^75^, designated in parenthesis are actual lengths of 3′UTRs based on 3′ RACE (Fig. S1C). ^^^Predicted number of miRNA binding sites (http://www.microrna.org/)^41^ within each 3′UTR as transcribed (3′ RACE). The value in the parenthesis represents the number of binding sites for miRNAs previously found to be expressed in *Drosophila* S2 cells^76^. (**C**) The 3′UTR of the reporter greatly affected repression (ratio of NT/T for luciferase activity). Dual-luciferase assay was used to determine repression. Normalization was carried out using firefly luciferase activity. (**D**) NT reporter expression and normalized ratio NT/T for luciferase activity show a statistically significant correlation. (**E**) Expression of the NT reporter varies greatly while T reporter expression shows little variation. (**F**) Correlation between several characteristics of our 3′UTR reporters and repression. All data are depicted as mean ± SD.

Using a previously defined reporter system targeted by the miRNA bantam in *Drosophila* S2 cells^11^ (Fig. 1A), we assessed the ability of the 3′UTR, CDS and 5′UTR to modulate miRNA-mediated repression. The reporter system includes a targeted (T) *Renilla* luciferase reporter that contains six target sites for the miRNA bantam in the 3′UTR and a non-targeted (NT) reporter containing reversed bantam sites, both of which are tightly controlled by the metallothionein promoter^37^. The 3′UTR has been implicated in regulation of translation and mRNA stability^28,38–40^. In order to assess how different 3′UTRs modulate repression of our reporter system we inserted the 3′UTR of several different genes from *Drosophila melanogaster* downstream of the miRNA target sites or reversed sites in our reporters (Fig. 1B). The 3′UTRs of these genes were chosen because they represent a wide range of cellular functions and have widely varying lengths (Fig. 1B). None of the selected 3′UTRs contained a predicted bantam binding site^41^. We used a dual luciferase assay to determine repression of each reporter, as done previously^11^. Strikingly we observed a wide variation in the magnitude of translational repression, with the reporter containing the GAPDH 3′UTR being repressed over 100-fold while the Cad87 3′UTR reporter was repressed less than 5-fold (Fig. 1C). There was very little variation in the magnitude of mRNA-degradation and no significant correlation between mRNA-degradation and translational repression (Fig. S1A and S1B). The expression of the NT reporter for each 3′UTR, however, correlated strongly with the observed translational repression, r = 0.904 and r_s_ = 0.964 (Fig. 1D). We define the variation in NT reporter expression as ‘translatability’. The NT reporter expression varied over two orders of magnitude. While repression by bantam was abrogated upon co-transfection with a bantam antagomir, the 3′UTR dependent effect on translatability was still observed with reporters lacking the bantam target sites or the control sequence (Fig. S2). Additionally there was a significant correlation between mRNA expression of our NT 3′UTR reporters and translatability (Fig. S3A and S3B). As such, our results indicate that both mRNA expression and the translation rate of our reporter constructs are altered by the 3′UTR. Differences in the protein expression levels (Fig. 1E), however, exceeded observed differences at the mRNA level (Fig. S1A). Interestingly, we saw less than two-fold variation in the protein expression of the targeted reporter for each 3′UTR (Fig. 1E). Together these data show that the magnitude of miRNA-mediated translational repression is dependent on the translatability of the target mRNA.

We did not observe correlation between repression or translatability and several other characteristics of the 3′UTRs (Fig. 1F). We performed 3′ rapid amplification of cDNA ends (3′RACE) to determine the length of each 3′UTR as expressed. While there was not a statistically significant correlation between 3′UTR length and translational repression for all our reporters, (Fig. 1F), we did observe a negative correlation for several 3′UTR reporters (Fig. S1D,E). It is well known that the presence and length of the poly(A)-tail can influence translation^38,42^ and multiple models have indicated that the progressive shortening of the poly(A)-tail is one of the mechanisms through which miRNAs exert translational repression on their targets^43,44^. Using a commercially available poly(A)-tail length assay, we determined the poly(A)-tail length of both the NT and T reporters. Consistent with previous work in *Drosophila* S2 cells we did not observe a shortening of the poly(A)-tail in our miRNA-target reporters (Fig. S3C–E)^11^. To test whether the presence of a poly(A)-tail is needed for the observed correlation between translatability and repression of our reporters we replaced the poly(A)-tail with the histone H3 stem loop (H3-SL). The switch from the poly(A)-tail to the H3-SL created an additional set of variations in the magnitude of miRNA-mediated repression (Fig. S4A). We have again observed high variability of NT reporter expression compared to T reporter at the protein level (Fig. S4B and C). We again did not observe any significant effects on mRNA ratio between NT and T reporters (Fig. S4D). While there was a clear difference between the translatability of reporters terminated by a poly(A)-tail or the H3-SL (Fig. S4B), the presence or absence of poly(A)-tail did not influence the correlation between translatability and miRNA-mediated repression (Fig. 1D and S4E). This result indicates that the observed correlation of the magnitude of miRNA-mediated repression and translatability is independent of the poly(A)-tail.

### miRNA-mediated repression is modulated by changes in codon optimality and 5′UTR

**Figure 2:**
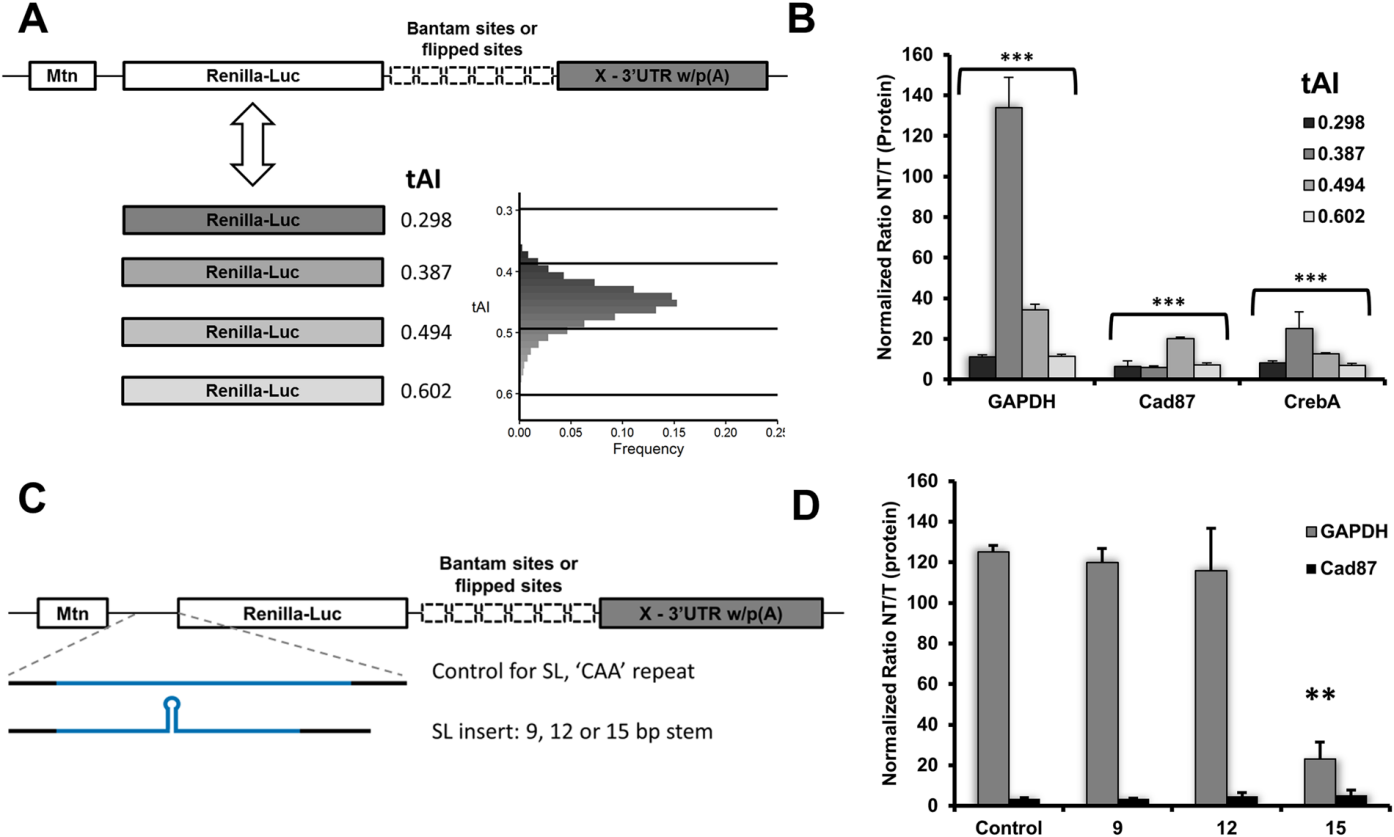
Codon optimality and 5′UTR elements modulate miRNA-mediated repression. The coding sequence of *Renilla* luciferase in the 3′UTR reporters for GAPDH, CrebA and Cad87 was modified to increase or decrease the codon optimality. (**A**) The tAI (tRNA Adaptation Index) of the original *Renilla* luciferase along with the modified versions is described. The inset histogram describes the frequency of tAI across all *D. melanogaster* genes. (**B**) Repression as determined by dual-luciferase assay was robust for reporters containing *Renilla* luciferase with moderate codon optimality, 0.387 and 0.494 tAI. ***p < 0.001 by *ANOVA*. Stem-loop (SL) structures were inserted into the 5′UTR of the reporters containing the GAPDH and Cad87 3′UTR. (**C**) Schematic describing the 5′UTR inserts used in panel D. (**D**) Repression was reduced for the GAPDH reporter containing the 15-bp SL inserted in the 5′UTR, while repression of the Cad87 reporter was unaffected by changes to the 5′UTR structure. **p < 0.01by *t*-test. All data are depicted as mean ± SD.

Having observed a wide variation in the translatability of our reporters simply by changing the 3′UTR, we wanted to explore how changes in other parts of the mRNA could affect miRNA-mediated repression. We changed the codon optimality of *Renilla* luciferase in our GAPDH, CrebA and Cad87 reporters. The original coding sequence (CDS) of *Renilla* had an optimality of 0.387 by the tRNA adaptation index (tAI)^33^. This value is close to the median tAI of all *D. melanogaster* genes (Fig. 2A). To sample a range of different tAI, values we created reporters with tAI of 0.602, 0.494 and 0.298 (Fig. 2A). Reducing the codon optimality of *Renilla* luciferase within our reporters reduced expression of the NT and T reporters by nearly three orders of magnitude (Fig. S5B and C). Interestingly, the repression of the GAPDH, CrebA and Cad87 3′UTR reporters was affected differently by changes to codon optimality (Fig. 2B). The GAPDH and CrebA reporters had peak repression when using the 0.387 tAI CDS, while the Cad87 reporter had peak repression using the 0.494 tAI CDS. All reporters showed reduced repression with the highest and lowest codon optimalities, 0.298 and 0.602. Consistent with our previous results, we found that the repression and expression of the reporters were affected differently by changes to the codon optimality depending on which 3′UTR was present (Fig. S5). This result again highlights the interaction between the 3′UTR and translatability. Together, the 3′UTR and codon optimality determine the magnitude of miRNA-mediated repression.

To further examine how mRNA elements may influence miRNA-mediated repression we assayed effects of the 5′UTR on miRNA-mediated repression. Recent evidence supports a model in which the miRISC inhibits translation via targeting the helicase eIF4A^45–47^ during 5′UTR scanning in search for a start codon. It is also known that introduction of secondary structure into 5′UTR affects initiation rate and total protein output^20,21,27^. Therefore, we inserted stem-loop structures into the 5′UTR of our GAPDH and Cad87 3′UTR reporters, which showed maximal and minimal miRNA-mediated repression respectively (Figs 1C and 2C). As seen in previous studies, insertion of the stem-loop structures in the 5′UTR greatly reduced reporter expression measured by luciferase activity^20,21,27^ (Fig. S6A and B). Interestingly, we found that the addition of stem-loop structures had no effect on repression of the Cad87 3′UTR reporter, which was minimal with the control insert (Fig. 2D). For our GAPDH 3′UTR reporter, which showed maximal repression with the control insert, we observed reduced repression upon insertion of a 15 bp stem-loop (Fig. 2D). In addition to testing the effect of specific 5′UTR elements, we also made reporters with 5′UTRs from *Drosophila* mRNAs. In particular, we paired the 3′UTR of the reporters described in Fig. 1 with their cognate 5′UTR. We again observed wide variation in the magnitude of repression, consistent with similar previous studies (Fig. S6C)^48^. To test the effect of short upstream ORFs on miRNA-mediated repression we inserted a short sequence coding for hemagglutinin-A epitope 55 nt upstream of the *Renilla* luciferase start site. The introduction of this short uORF in the GAPDH 3′UTR reporter increased repression by two fold (Fig. S6E). These results should be taken with caution, however, since translation of uORFs usually leads to activation of mRNA surveillance mechanisms. These events usually result in efficient and targeted mRNA decay of mRNAs with translated uORFs^17,24–26^. Therefore, it is not surprising that we observed great reduction in protein output of the downstream encoded luciferase reporter (Fig. S6F). Our data on 5′UTR structure and codon optimality in the context of different 3′UTRs indicate a connection between translatability and magnitude of miRNA-mediated translational repression.

### Translation efficiency is a determinant of miRNA-mediated repression

**Figure 3:**
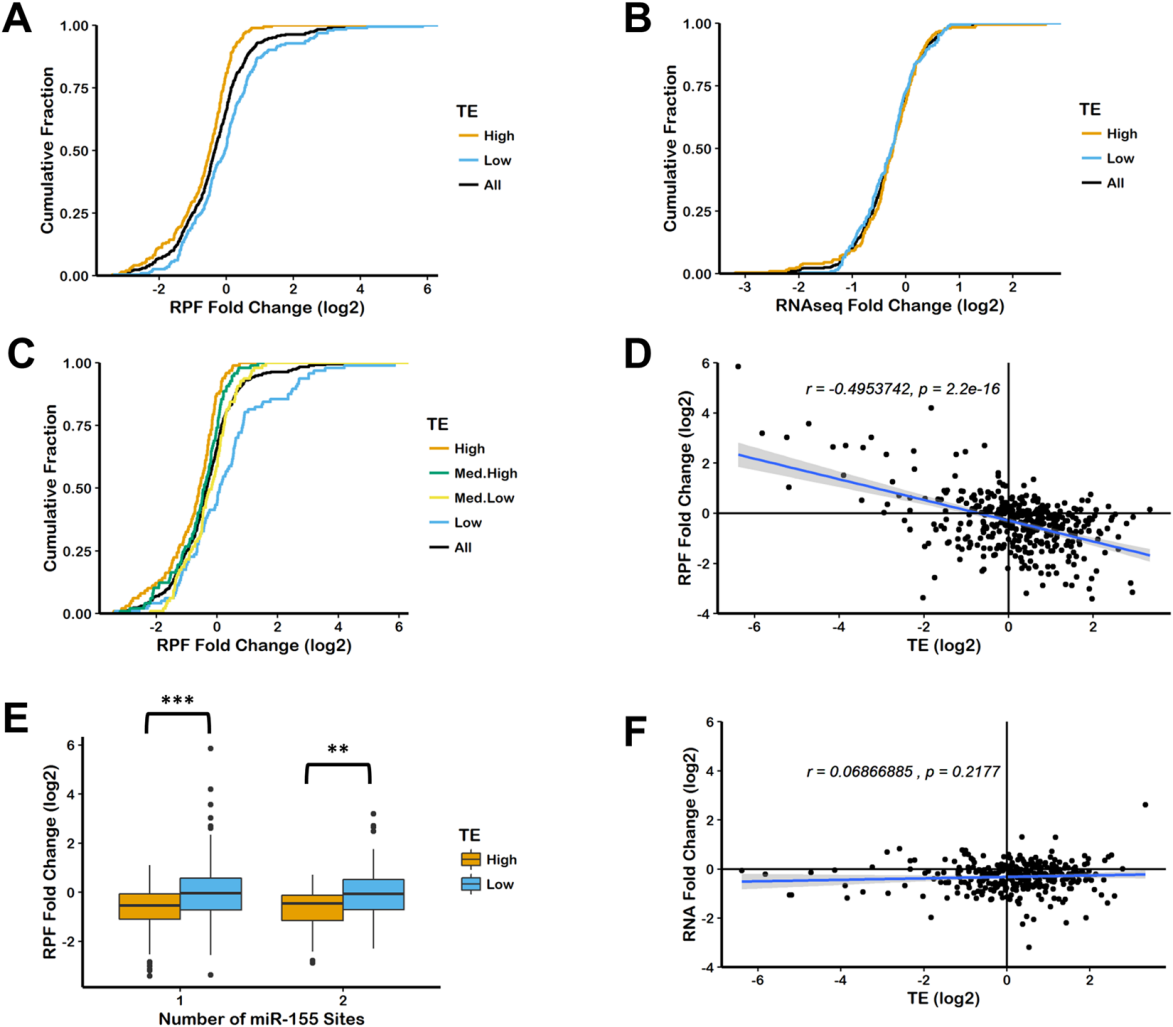
Translation efficiency is a determinant of the magnitude of miRNA-mediated translational repression but not RNA degradation of endogenous miRNA targets. Cumulative distributions of fold change of RPF (ribosome protected fragments), (**A**,**C**) or fold change of RNA, (**B**) for all miR-155 predicted targets (http://targetscan.org/) in data from Guo *et al*., 2010. Fold change is calculated as the log2 normalized RPF or RNAseq reads for miR-155 transfected divided by mock transfected. TE for each transcript in the absence of miR-155 (mock transfection) was calculated by normalized RPF divided by normalized RNAseq reads. All miR-155 targets are binned by TE, above or below the median (“High” or “Low”), **A** and **B** or by TE quartiles, (**C)**(**D**,**F**) Correspondence between RPF fold change and TE, D, or RNA fold change and TE, **F**. **E** miR-155 targets were binned by the number of conserved and poorly conserved binding sites for miR-155 as well as TE. **p < 0.01, ***p < 0.001 by Kolmogorov–Smirnov test.

While reporter studies are valuable for understanding the mechanism of miRNA-mediated translational repression and mRNA deadenylation and degradation^11,35,45,47–51^, they might be limited since they study a relatively small number of targeted messages in controllable *in viv*o or *in vitro* conditions. MicroRNAs in living cells act on hundreds of endogenous genes which have more varied mRNA sequences than reporters. Moreover, cellular physiology is under constant change due to the complex level of transcriptional, translational and post-translational control, which are influenced by developmental, environmental and other physiological cues. In order to test the generality of our reporter studies, we turned to whole genome analysis of miRNA targets in HeLa cells^6^. The most striking observation made using our reporters is the strong correlation seen between translatability and repression. We compared fold change of ribosome protected fragments (RPF) for miR-155 targets following mock transfection or miR-155 transfection with translation efficiency of the target message in the absence of the miRNA (mock transfection). Translation efficiency (TE) is the ratio of RPF and RNA abundance determined by ribosome profiling and RNA-seq^52^. We consider TE as a good proxy for translatability. Comparison of TE and fold change of RPF revealed a strong interaction (Fig. 3A,C and E, Fig. S7). We binned all miR-155 targets by TE, either above or below the median TE or by quartiles. Transcripts with high TE were more repressed than those with low TE (log_2_ of RPF median fold change: High_TE_ = −0.486, Low_TE_ = −0.044, All miR-155 targets = −0.346). Separating miR-155 targets by TE quartile produced similar results (High = −0.588, Med.High = −0.419, Med. Low = −0.185, Low = 0.093). Further stratifying the miR-155 targets into messages containing a 6-mer, 7-mer-a1, 7-mer-m8 and 8-mer binding sites revealed that this interaction is not dependent on the type of miRNA-target pairing that is present (Fig. S8). These results were consistent with those of miR-1 transfection (Fig. S9). Interestingly, TE had no influence on miRNA-mediated mRNA decay (Fig. 3B and F). Since a correlation between translational repression (Fold Change of RPF) and mRNA-decay (Fold Change of RNAseq) had been shown previously^6,53^, the observation that TE influences translational repression but not mRNA-decay is surprising. This result suggests that the correlation between translational repression and mRNA-decay seen previously might also be dependent on TE. This was confirmed by nonlinear model fitting where the Pearson correlation coefficient between measured and predicted RPF fold change was much improved (0.44 vs 0.74) when TE was available as a variable in addition to the measurements of mRNA levels (Fig. S10). UTR-related variables (like length or MFE) did not improve the basic model.

Using the same approach, we analyzed recent data of miR-155 induced response in lipopolysaccharide (LPS) stimulated B-cells (Fig. S11). miR-155 is induced upon LPS stimulation in primary macrophages, dendritic cells, B and T cells^54–56^. Compared to exogenous expression of miR-155 in Hela cells, increased levels of miR-155 during LPS response is required for both translational and transcriptional activation and differentiation of B and T cells to cells characterized by production of IgM and switched antigen-specific antibodies^57,58^. Results from our analysis of this environmentally induced miRNA response further support our earlier observations and the correlation between translation efficiency and the magnitude of miRNA-mediated repression. Transcripts with high TE were more repressed than those with low TE (Fig. S11). This trend was observed at multiple time points and was specific for miR-155 targets (Fig. S12). Additional analysis of gene ontology groups (Fig. S13) identified similarly enriched functions for groups of genes selected by higher-than-median TE values or, independently, the significant level of repression (RPF FC values below −0.25), which independently implies the correlation between these two variables.

Having previously shown interactions between mRNA characteristics such as codon optimality and 5′UTR structure with miRNA-mediated repression we sought to study these interactions globally using ribosome profiling data. Interestingly, we did not observe any influence of 3′UTR length, transcript length, tAI, 5′UTR structure or 3′UTR structure on miRNA-mediated repression (Fig. S9, 14 and 15). We also did not find correlation between miRNA-mediated mRNA degradation or translational repression with global measurements of mRNA half-lives^59^ (Fig. S16). However, TE, especially in combination with mRNA degradation, was predictive of the magnitude of miRNA-mediated repression.

## Discussion

By using a systematic approach, we have revealed several mRNA elements capable of modulating miRNA-mediated repression. Our observations suggest efficient translational repression by the miRISC depends on the translation efficiency of the target.

Using luciferase reporters in *Drosophila* S2 cells we observed a strong interaction between the 3′UTR and the magnitude of repression. This result was largely driven by 3′UTR dependent differences in the translatability of the reporters. Translatability is likely the output of different mRNA characteristics such as *cis* and *trans* factors that modulate translation rate and mRNA stability. 3′UTR characteristics such as GC content and structure could not explain this variation. While there was not a significant correlation between 3′UTR length and translatability there was a trend for a few of the 3′UTRs tested. This observation is supported by analysis of several whole-genome studies of miRNA-mediated repression which showed messages containing shorter 3′UTRs are more repressed than messages with long 3′UTRs^60^. Beyond structural features of the 3′UTR, the presence of RBPs or miRNAs likely influences the translatability of some of the 3′UTRs tested. Several RBPs have been shown to modulate miRNA-mediated repression^13–15^. We cannot exclude that additional binding of RBPs or other miRNAs to the assayed 3′UTRs may also affect the translation rate but we assume that these effects are preserved in both targeted and non-targeted reporter. In order to thoroughly address the possibility of RBPs modulating the miRNA-mediated repression of our 3′UTR reporters we need a more thorough understanding of which RBPs are bound to those 3′UTRs and how those RBPs functionally interact with the miRISC. Beyond RBPs many of the miRNAs that are predicted to target the 3′UTRs are either not expressed or expressed at a very low level (Table S2).

Upon changing the codon optimality of our miRNA-targeted reporters we observed variation in repression. Reporters with very high or low codon optimality were poorly repressed compared to reporters with intermediate optimality. This was true for all reporters but there were differences in the expression and repression of the reporters that were 3′UTR dependent. This observation suggests some interplay between the 3′UTR and codon optimality, which is consistent with recent report that the stability of maternally deposited mRNAs in zebrafish is regulated by the combined effect of codon optimality and 3′UTR length^61^. Furthermore, the variability of miRNA-mediated repression caused by changes in codon optimality indicates again that translatability has an influence on the magnitude of miRNA-mediated repression. Paradoxically, the reporters with the highest codon optimality and highest expression were poorly repressed. A possible explanation for this finding can be that the miRISC is most effective at inhibiting the translation of efficiently translated mRNAs. While codon optimality is thought to influence the rate of translation elongation, the overall rate of translation includes the rate of initiation and termination. Our results suggest that when one of these rates are changed, but not the others, the efficiency of miRNA-mediated repression is altered. An intriguing possible explanation for the effects of the various 3′UTRs on miRNA-mediated repression is that each 3′UTR is affecting either the initiation or elongation rate. This could help to explain the interplay between codon optimality and the 3′UTR, in one potential scenario the 3′UTR is increasing or decreasing the initiation rate which could enhance or repress the effects of changing the codon optimality. For instance, the overall translation rate of a message with very slow initiation may be less affected by increasing codon optimality. This balance between initiation rate and elongation rate would be reflected as a change in TE. Messages with more balanced translation would have higher TE, and as we have shown messages with higher TE are more repressed by the miRISC.

Our analysis of previously published ribosome profiling data revealed TE to be a determinant of miRNA-mediated repression. This observation was true for several miRNAs across multiple cell lines. This finding was consistent with our 3′UTR reporter study where we observed a correlation between the translatability of the reporter and its miRNA-mediated repression. In the context of what is known about miRNA-mediated repression these findings make sense. Since the miRISC inhibits translation, messages that are translated well should show the most repression. These findings and those made using reporters help to explain the wide variation seen in the magnitude of translational repression using various reporters and in whole-genome studies of miRNA function. We were unable to find any correlation between miRNA-mediated repression and various mRNA characteristics within the ribosome profiling data. We suspect that since each transcript possesses many varied features (tAI, CDS length, transcript length, 3′UTR length, 5′UTR length, 5′UTR structure, binding sites for miRNAs, RBPs, the presence of uORFs, etc.) that the interactions of any one of this features and miRNA-mediated repression are subtle due to this complexity. Perhaps with more knowledge of the interactions of these elements with each other and the miRISC a more sophisticated model could be built to predict the magnitude of miRNA-mediated repression.

**Figure 4:**
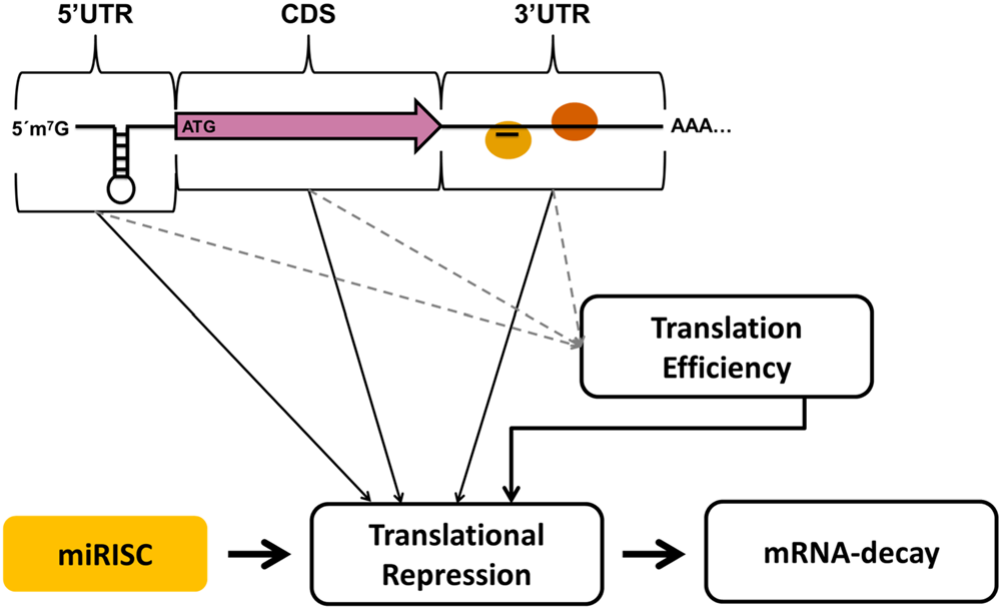
Model: Translation efficiency and mRNA elements influence the magnitude of miRNA-mediated repression. Multiple mRNA elements along with translation efficiency influence the magnitude of miRNA-mediated repression. MiRNA targets with relatively high TE will be more robustly repressed than targets with relatively low TE. Some mRNA elements may directly influence the magnitude of miRNA-mediated repression while others may have an indirect effect my changing TE.

Our data also help to further define the mechanism of miRNA-mediated repression. When considering our results with kinetic analyses of miRNA-mediated repression, which have previously shown translational repression preceding mRNA-decay^5,7,10,11,36^; a model for miRISC function can be generated in which translational repression precedes mRNA decay, and while the magnitude of translational repression is dependent on TE the magnitude of mRNA-decay is not (Fig. 4). Recently it has been observed that miRNA targeted mRNAs can be degraded co-translationally^51^. This observation directly links the translation status of a miRNA target with its decay. We suspect that the magnitude of mRNA-decay is dependent on the susceptibility of the message to deadenylation and decay which may vary from cell-to-cell based on the abundance of decapping/deadenylation factors and from message-to-message based on the presence of *cis* and *trans* elements that affect this process. This model therefore allows for a scenario in which an mRNA may serve as an effective target for translational repression because of its TE but not for mRNA-decay or vice versa. Our model fits well with the hypothesis that miRNAs serve dual functions: to induce robust changes in gene-expression during development and other biological processes or small changes in gene-expression to balance stochastic gene-transcription^1^.

Finally, our analysis of endogenous miRNA targets highlights the difficulty of studying the effects of mRNA elements and characteristics such as translation elongation and initiation rates on miRNA-mediated repression at the whole genome level. Each message possesses so many variables that the effects of any one variable on miRNA-mediated repression are masked. Additionally, biological processes are under complex control at different molecular levels. An example of this can be seen during activation of immune cells where changes in the transcriptome and proteome results from epigenetic, transcriptional and post-transcriptional regulation, controlled in part by miRNAs^62–64^. Due to the complexity of these changes, and interactions between key factors at each regulatory level, it will be hard to tease apart the direct influence of one specific factor, even for post-transcriptional regulation alone^62^. Therefore, one approach to study the detailed mechanics of miRNA-mediated repression is to use reporters in appropriate cells types. More comprehensive analysis of the effects of *cis* and *trans* elements of the mRNA on miRNA-mediated repression will be essential for pinpointing the mechanism of miRNA-mediated repression and refining models of effective miRNA target prediction.

## Materials and Methods

### Construction of Reporters

All primers used for cloning can be found in the Supplemental Table 1. *Renilla* luciferase along with either six bantam sites or six flipped bantam sites were PCR amplified from pMT-DEST48-HID and pMT-DEST48 FLP^11^ using the *Renilla* forward and HID/FLP overlap reverse primers. This PCR product was used in overlap PCR to construct the 3′UTR reporters used in the study. The 3′UTRs were amplified by the primers designated in Supplemental Table 1 (for example: GAPDH forward overlap and reverse). The forward primer for each 3′UTR contained a 25–26 nt sequence complimentary to the HID/FLP overlap reverse primer used above. The PCR product for each 3′UTR and the PCR product containing *Renilla* luciferase and the targeted/non-targeted bantam sites were used in overlap PCR with a *Renilla* forward primer and a reverse primer specific for each 3′UTR. This product was then cloned into pENTR/D-TOPO (Invitrogen) per the manufacturer′s protocol. For the Rpl32 3′UTR reporter a reverse primer containing the Rpl32 3′UTR (Rpl32 reverse) was used to add the Rpl32 3′UTR to the PCR product containing *Renilla* luciferase and the bantam sites. The constructs were confirmed by Sanger sequencing and subsequently cloned into the pMT-DEST48-p(A)sΔ plasmid using LR-Clonase (Invitrogen). These constructs were confirmed by Sanger sequencing. The pMT-DEST48-p(A)sΔ plasmid was made by site directed mutagenesis to remove the SV40 p(A) signal from pMT-DEST48 (Invitrogen) using the SV40 p(A)s mutagenesis forward and reverse primers.

To make 3′UTR reporters terminated by the H3 stem-loop we first constructed pMT-DEST48-H3. The pMT-DEST-48-p(A)sΔ plasmid was digested with *PmeI* and subsequently ligated with oligonucleotides H3-SL oligonucleotide 1 and 2. The 3′UTR for GAPDH, Hsp70, Alpha-tubulin, beta-tubulin and CrebA were amplified with the forward primer used for the initial cloning of the 3′UTR and a reverse primer located upstream of the native p(A) signal (for example: GAPDH-p(A) reverse). Overlap PCR was performed as described above to fuse *Renilla* luciferase and the bantam sites with the 3′UTR and this product was subsequently cloned into pENTR/D-TOPO (Invitrogen) per the manufacturer′s protocol. The constructs were confirmed by Sanger sequencing and subsequently cloned into the pMT-DEST-48-H3 plasmid using LR-Clonase (Invitrogen).

To make 3′UTR reporters with codon modified *Renilla* coding sequences we digested the expression plasmid for each 3′UTR reporter (for example: pMT-pAs-GAPDH) with *NcoI* and *KpnI* to remove *Renilla* luciferase. The digest was resolved on an agarose gel and the appropriate band was excised and purified. This product was then ligated with coding sequence for *Renilla* luciferase with a tAI of 0.602, 0.494 or 0.298. The *Renilla* luciferase coding sequence was synthesized by Invitrogen (coding sequence shown in Supplementary Information) and was digested with *NcoI* and *KpnI* prior to ligation. The resulting constructs were confirmed by Sanger sequencing.

To make insertions into the 5′UTR of our 3′UTR reporters we digested the desired 3′UTR reporter with *SacII*. The digest product was ligated with oligos containing the 5′UTR insert (control and stem-loops of 9, 12, 15 and 18 bps as well as the uORF control or uORF). The resulting constructs were confirmed by Sanger sequencing.

To insert the cognate 5′UTR for each 3′UTR reporter described above we digested the vector containing the 3′UTR reporter (pMT-DEST48) with *MscI* and *NcoI*. This digest removed the 5′UTR present in the vector. The 5′UTRs to be inserted were PCR amplified from S2 cell cDNA with primers described in Table S1. The forward primer for each contained 20 nt corresponding to the transcription start site and flanking bases (5′ CCAATGTGCATCAGTTGTGG 3′) that were removed from the vector by the digest. The PCR product was digested with *NcoI* and the product was ligated into the digested plasmids described above.

The 3′UTR reporters were made without bantam target sites or control sequences by overlap PCR using primers designed to amplify the 3′UTR and primers designed to amplify *Renilla* luciferase described in Table S1. This product was then cloned into pENTR/D-TOPO (Invitrogen) per the manufacturer’s protocol. The constructs were confirmed by Sanger sequencing and subsequently cloned into the pMT-DEST48-p(A)sΔ plasmid using LR-Clonase (Invitrogen).

### Transfection and Luciferase Assay

For most experiments 100 ng of the 3′UTR reporter as well as 100 ng each of pMT-firefly-luciferase, pAC-bantam and 200 ng of pMT-bantam^11^ were transfected into one well of a six well dish containing drosophila S2 cells (Invitrogen). The transfection was performed using Effectene (Qiagen) per the manufacturer’s protocol. Four hours after transfection the media was removed and replaced with media containing 500 µg/mL CuSO_4_ to induce expression of the 3′UTR reporter as well as firefly luciferase and bantam. Two hours post induction the media was removed and the cells were briefly washed with media containing 50 µg/mL bathocuproine disulfonate (BCS). Following the wash 2 mL of media containing 50 µg/mL BCS was added to each well and the cells were resuspended and split between two wells in separate 12-well plates. The cells were then allowed to incubate for 16 hours. After 16 hours one of the 12-well plates was harvested for measurement of luciferase activity while the other was used to isolate RNA, see below. For the luciferase assay the culture media was removed from the cells and 250–400 µL of 1x Passive Lysis Buffer (Promega) was added. The cells were lysed for 15 minutes while rocking at room temperature. The lysate was cleared by centrifugation at 21,000 g for 1 minute. An aliquot of the lysate was then used to measure firefly and *Renilla* luciferase activity using the Dual Glo Luciferase System (Promega) and the Glomax plate reader (Promega) per the manufacturer’s instructions. All luciferase assays were performed in triplicate. *Renilla* luciferase activity was normalized to firefly luciferase.

For co-transfection of antagomirs, the transfection was carried out in a 12-well format. Cells were transfected with 50 ng of the 3′UTR reporter as well as 50 ng each of pMT-firefly-luciferase, pAC-bantam and 100 ng of pMT-bantam or for antagomir treated cells 50 ng of the 3′UTR reporter as well as 50 ng pMT-firefly-luciferase, 200 nM batnam antagomir (IDT) and 150 ng of pMT-CFP to maintain the DNA concentration. The cells were induced and harvested as described above.

### RNA Analysis

RNA was extracted from S2 cells using either Ribosol (Amresco) or SIGMA RNA mini-prep per manufacturer’s protocol. Isolated RNA was DNase treated with Turbo DNase (Ambion) prior to cDNA synthesis. For cDNA synthesis 5x iScript Supermix (Bio-Rad) was used per manufacturer’s protocol. Quantitative PCR was performed with primers targeting *Renilla* luciferase or firefly luciferase, Supplemental Table 1. For 3′RACE: cDNA was synthesized using 3′RACE RT primer and 5x iScript Select (Bio-Rad) per manufacturer’s protocol. First round PCR was performed with *Renilla*-tail forward and 3′RACE External Amp primers. Second round PCR was performed with the overlap-forward primer for each 3′UTR being amplified (for example: GAPDH forward overlap) and 3′RACE amplification primer. The PCR products were resolved on an agarose gel. Prominent bands were excised and sequenced by Sanger sequencing. For qPCR of mature miRNAs we followed the protocol described previously^65^. The primers used for this analysis are described in Table S3.

For analysis of p(A)-tail length we used the Poly(A) Tail-Length Assay Kit from Thermo-Fisher. The assay was performed per the manufacturer’s protocol. The primers used are described in Table S1: Hsp70a R, GAPDH R2 and HID/FLP F.

### Cell Culture

Drosophila Schneider 2 (S2) cells were maintained in High Five Serum Free Media (Invitrogen) supplemented with 1× penicillin, streptomycin and glutamine (PSG) (Gibco) and 20 mM glutamine (Gibco).

### Analysis of Ribosome Profiling and RNA-seq

The accession number for ribosome profiling and RNA-seq data used in this study is GSE22004. Fold change of RPF and RNA-seq was calculated as described in Guo *et al*.^6^. Translation efficiency (TE) was calculated using RPF and RNA-seq rpkM from mock transfection, TE = (rpkM_RPF_/rpkM_RNA_). We obtained transcript, CDS and 3′UTR length for human genes from Ensembl using BioMart^66,67^. mRNA half-lives were obtained from 5′-bromo-uridine (BrU) immunoprecipitation chase-deep sequencing analysis of HeLa mRNAs^59^. miR-155 or miR-1 targets were predicted using TargetScan^60,68,69^. The tRNA adaptation index for each gene was calculated using CodonR (https://github.com/dbgoodman/ecre_cds_analysis/tree/master/codonR). For this analysis the CDS of all human or *Drosophila* genes was obtained from the UCSC Table Browser^70^ and the tRNA gene table for human or *Drosophila* was obtained from the GtRNAdb^71^. Analysis of ribosome profiling and RNA-seq data was performed in R 3.2.4^55^ using packages ggplot2^56^ and extrafont^57^. Scripts in R used for analysis are available at the Github repository under MIT license (https://github.com/freesci/translationefficiency). Gene ontology terms enrichment assessed with FunRich^72^.

### Model fitting

All genes with complete information (mRNA and RPF levels) from miR-155 repression experiment were further analyzed for the relationships between fold change, TE and other variables. In addition to statistics collected above, we have calculated MFE of both UTRs using Vienna package^73^ and normalized against sequence length using the approach described by Trotta^74^. These variables were later imported into Eureqa software from Nutonian that dynamically fits a variety of equations into the data. Several experiments were done using different approaches to scoring function, from absolute error (the software default) to R^2^ coefficient of determination which was chosen for the final plots.

## Electronic supplementary material

Supplementary Information
