# Supplementary material for: Translation efficiency is a determinant of the magnitude of miRNA-mediated repression
Source: Sci Rep. 2017 Nov 2;7:14884. doi: 10.1038/s41598-017-13851-w (PMC5668238; doi:10.1038/s41598-017-13851-w)
Supplement: Supplementary file 1 — Supplementary Information [file 41598_2017_13851_MOESM1_ESM.doc]

Supplementary Information for

**Translation efficiency is a determinant of the magnitude of miRNA-mediated repression**

Kyle A Cottrell, Pawel Szczesny, Sergej Djuranovic*

*correspondence to: sergej.djuranovic@wustl.edu

**This PDF file includes:**

*Renilla* Luciferase Coding Sequences

Figure S1 – S16

Table S1-S3

­*Renilla* Luciferase Coding Sequences

>Ren_luc_0.298

ATGGGGACTTCTAAAGTATATGACCCTGAACAAAGAAAAAGAATGATAACTGGGCCTCAATGGTGGGCAAGGTGTAAACAAATGAATGTACTAGACTCATTTATAAATTATTATGACTCTGAAAAGCATGCAGAAAATGCAGTAATATTTTTACATGGGAATGCAGCATCATCTTATCTATGGAGACATGTAGTTCCTCATATAGAACCTGTAGCGAGGTGTATAATTCCTGACTTAATAGGGATGGGGAAGTCAGGTAAATCTGGGAACGGTTCTTATAGGCTATTAGACCATTATAAATATCTAACTGCATGGTTTGAATTACTAAATCTACCTAAAAAGATAATATTTGTAGGGCATGACTGGGGGGCATGTCTAGCATTTCATTATTCTTATGAACATCAAGACAAAATAAAGGCAATAGTACATGCAGAATCTGTAGTAGACGTAATAGAGTCATGGGACGAATGGCCTGACATAGAAGAGGACATAGCACTAATAAAATCAGAAGAAGGTGAAAAGATGGTATTAGAAAATAATTTCTTTGTAGAAACTATGCTACCTTCAAAAATAATGAGAAAGTTAGAACCTGAAGAATTTGCAGCATATTTAGAACCTTTTAAAGAGAAAGGAGAAGTAAGAAGGCCTACTTTATCATGGCCTAGAGAAATACCTTTAGTAAAAGGGGGTAAACCTGACGTAGTACAAATAGTAAGAAATTATAATGCATATCTAAGAGCATCTGACGACTTACCTAAAATGTTTATAGAGTCTGACCCTGGGTTCTTTTCAAATGCAATAGTAGAAGGGGCAAAAAAATTTCCTAATACTGAGTTTGTAAAAGTAAAAGGGCTACACTTTTCTCAAGAAGACGCACCTGACGAAATGGGGAAATATATAAAATCTTTTGTAGAGAGAGTATTAAAAAATGAACAATAA

>Ren_luc_0.387

ATGGGCACTTCGAAAGTTTATGATCCAGAACAAAGGAAACGGATGATAACTGGTCCGCAGTGGTGGGCCAGATGTAAACAAATGAATGTTCTTGATTCATTTATTAATTATTATGATTCAGAAAAACATGCAGAAAATGCTGTTATTTTTTTACATGGTAACGCGGCCTCTTCTTATTTATGGCGACATGTTGTGCCACATATTGAGCCAGTAGCGCGGTGTATTATACCAGACCTTATTGGTATGGGCAAATCAGGCAAATCTGGTAATGGTTCTTATAGGTTACTTGATCATTACAAATATCTTACTGCATGGTTTGAACTTCTTAATTTACCAAAGAAGATCATTTTTGTCGGCCATGATTGGGGTGCTTGTTTGGCATTTCATTATAGCTATGAGCATCAAGATAAGATCAAAGCAATAGTTCACGCTGAAAGTGTAGTAGATGTGATTGAATCATGGGATGAATGGCCTGATATTGAAGAAGATATTGCGTTGATCAAATCTGAAGAAGGAGAAAAAATGGTTTTGGAGAATAACTTCTTCGTGGAAACCATGTTGCCATCAAAAATCATGAGAAAGTTAGAACCAGAAGAATTTGCAGCATATCTTGAACCATTCAAAGAGAAAGGTGAAGTTCGTCGTCCAACATTATCATGGCCTCGTGAAATCCCGTTAGTAAAAGGTGGTAAACCTGACGTTGTACAAATTGTTAGGAATTATAATGCTTATCTACGTGCAAGTGATGATTTACCAAAAATGTTTATTGAATCGGACCCAGGATTCTTTTCCAATGCTATTGTTGAAGGTGCCAAGAAGTTTCCTAATACTGAATTTGTCAAAGTAAAAGGTCTTCATTTTTCGCAAGAAGATGCACCTGATGAAATGGGAAAATATATCAAATCGTTCGTTGAGCGAGTTCTCAAAAATGAACAATAA

>Ren_luc_0.494

ATGGGCACCTCGAAGGTGTACGATCCAGAGCAACGTAAGCGGATGATTACCGGCCCCCAGTGGTGGGCACGCTGCAAGCAAATGAACGTGCTAGACTCCTTCATCAACTACTACGATTCGGAGAAGCATGCGGAGAACGCCGTGATCTTCTTGCATGGTAATGCCGCCAGCAGCTACCTGTGGCGGCACGTTGTTCCCCACATTGAGCCCGTCGCCAGGTGCATTATCCCCGACCTGATTGGCATGGGTAAGAGTGGCAAGAGCGGTAACGGAAGCTACCGCTTGCTTGATCATTATAAATACCTGACCGCCTGGTTTGAGCTTTTGAACCTGCCCAAGAAGATCATCTTCGTGGGTCACGATTGGGGCGCGTGCCTTGCCTTCCATTACTCTTATGAGCACCAGGACAAAATAAAAGCCATTGTGCACGCCGAGTCCGTTGTGGACGTGATTGAGTCGTGGGATGAATGGCCCGACATTGAAGAGGATATTGCCTTAATCAAAAGCGAGGAAGGAGAAAAGATGGTGCTCGAGAATAACTTCTTCGTTGAGACCATGCTGCCCTCCAAGATCATGAGAAAGCTGGAACCTGAGGAGTTTGCCGCCTATCTTGAGCCCTTTAAGGAGAAGGGAGAGGTGAGGCGTCCAACACTGTCTTGGCCCCGCGAGATCCCGCTGGTGAAAGGTGGCAAACCCGATGTCGTGCAGATCGTGAGGAACTACAATGCGTATCTTCGTGCTTCGGACGATCTGCCCAAGATGTTCATCGAGTCGGATCCGGGATTTTTCTCCAATGCCATCGTGGAAGGCGCTAAGAAGTTTCCGAACACTGAGTTTGTGAAGGTGAAGGGTCTGCACTTCAGCCAAGAAGATGCACCGGACGAAATGGGTAAATACATTAAGAGTTTCGTCGAAAGGGTCCTCAAAAATGAGCAATAA

>Ren_luc_0.602

ATGGGCACCTCCAAGGTGTACGACCCCGAGCAGCGCAAGCGCATGATCACCGGCCCCCAGTGGTGGGCCCGCTGCAAGCAGATGAACGTGCTGGACTCCTTCATCAACTACTACGACTCCGAGAAGCACGCCGAGAACGCCGTGATCTTCCTGCACGGCAACGCCGCCTCCTCCTACCTGTGGCGCCACGTGGTGCCCCACATCGAGCCCGTGGCCCGCTGCATCATCCCCGACCTGATCGGCATGGGCAAGTCCGGCAAGTCCGGCAACGGCTCCTACCGCCTGCTGGACCACTACAAGTACCTGACCGCCTGGTTCGAGCTGCTGAACCTGCCCAAGAAGATCATCTTCGTGGGCCACGACTGGGGCGCCTGCCTGGCCTTCCACTACTCCTACGAGCACCAGGACAAGATCAAGGCCATCGTGCACGCCGAGTCCGTGGTGGACGTGATCGAGTCCTGGGACGAGTGGCCCGACATCGAGGAGGACATCGCCCTGATCAAGTCCGAGGAGGGCGAGAAGATGGTGCTGGAGAACAACTTCTTCGTGGAGACCATGCTGCCCTCCAAGATCATGCGCAAGCTGGAGCCCGAGGAGTTCGCCGCCTACCTGGAGCCCTTCAAGGAGAAGGGCGAGGTGCGCCGCCCAACCCTGAGTTGGCCACGCGAGATCCCCCTGGTGAAGGGCGGCAAGCCCGACGTGGTGCAGATCGTGCGCAACTACAACGCCTACCTGCGCGCCTCCGACGACCTGCCCAAGATGTTCATCGAGTCCGACCCCGGCTTCTTCTCCAACGCCATCGTGGAGGGCGCCAAGAAGTTCCCCAACACCGAGTTCGTGAAGGTGAAGGGCCTGCACTTCTCCCAGGAGGACGCCCCCGACGAGATGGGCAAGTACATCAAGTCCTTCGTGGAGCGCGTGCTGAAGAACGAGCAGTAA

**
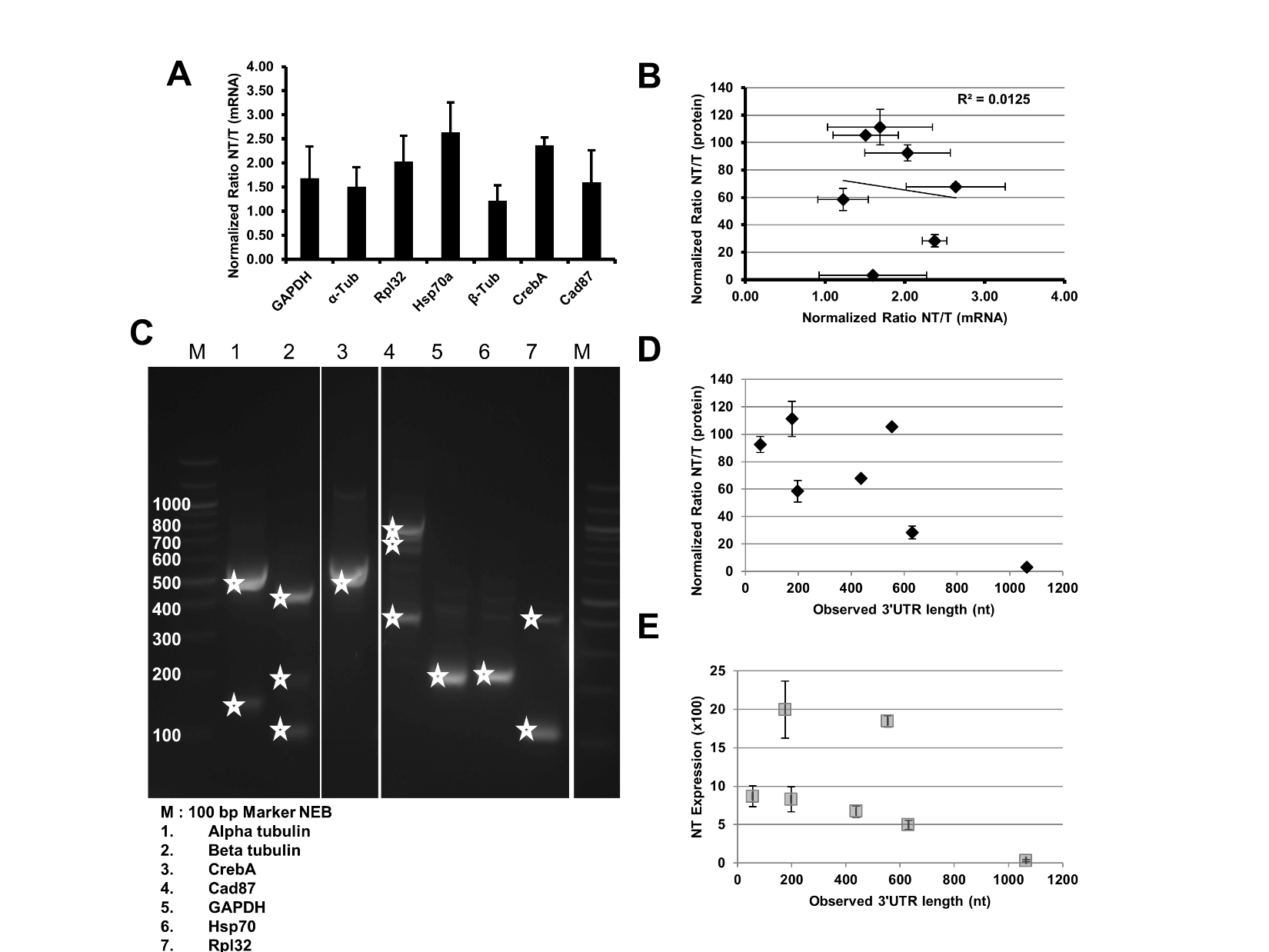
**

**Figure S1 | RNA-decay and 3’UTR length of *Renilla* Luciferase Reporters**

**A** The 3’UTR of the reporter minimally affected mRNA degradation. Quantitative RT-PCR was performed to determine fold RNA degradation (Normalized Ratio NT/T for mRNA). **B** There is no correlation between mRNA degradation and fold repression (normalized ratio of NT/T for luciferase activity). **C** Agarose gel of 3’RACE products for 3’UTR reporters used in this study. Stars indicate bands that were excised and sequenced. **D** Correspondence between the length of each 3’UTR (assigned by the most prominent band for each 3’UTR in **C**) as determined by sequencing of **C** and repression (normalized ratio of NT/T for luciferase activity) or NT Expression **E**. All data are depicted as mean ± SD.


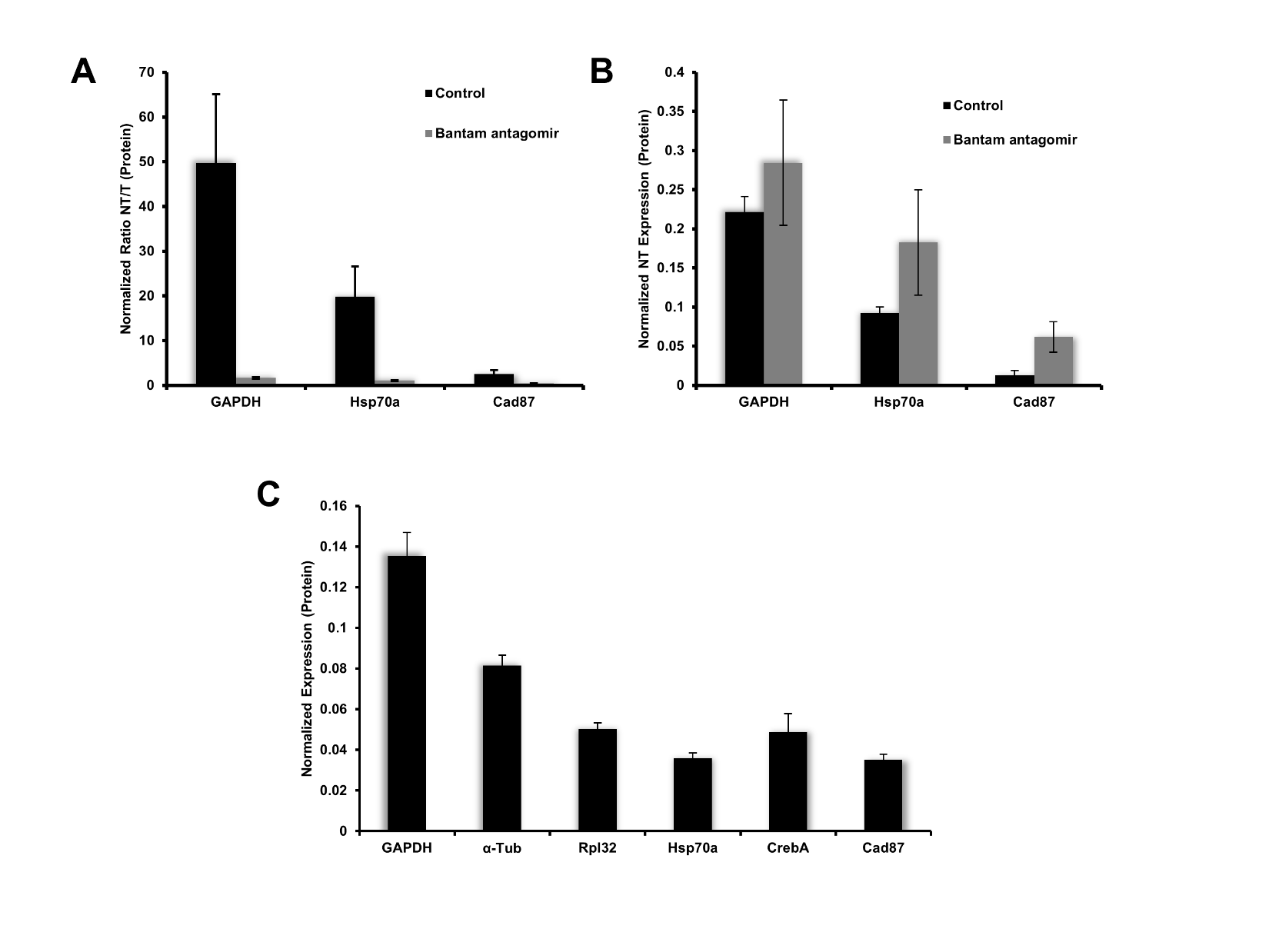


**Figure S2 | Bantam antagomir abrogates repression of 3’UTR reporters**

**A** Normalized ratio of NT/T expression as determined by luciferase assay. Co-transfection with bantam antagomir with a final concentration of 200 nM caused derepression of each 3’UTR reporter tested. **B** The normalized NT expression for the reporters in panel **A** is shown. There is no significant increase in expression upon co-transfection with bantam antagomir. **C** The bantam T/NT sites from the reporters in Figure 1A were removed and the 3’UTR was directly fused to the Renilla luciferase coding sequence. The expression of these new 3’UTR reporters is shown in panel **C**. All data are depicted as mean ± SD.


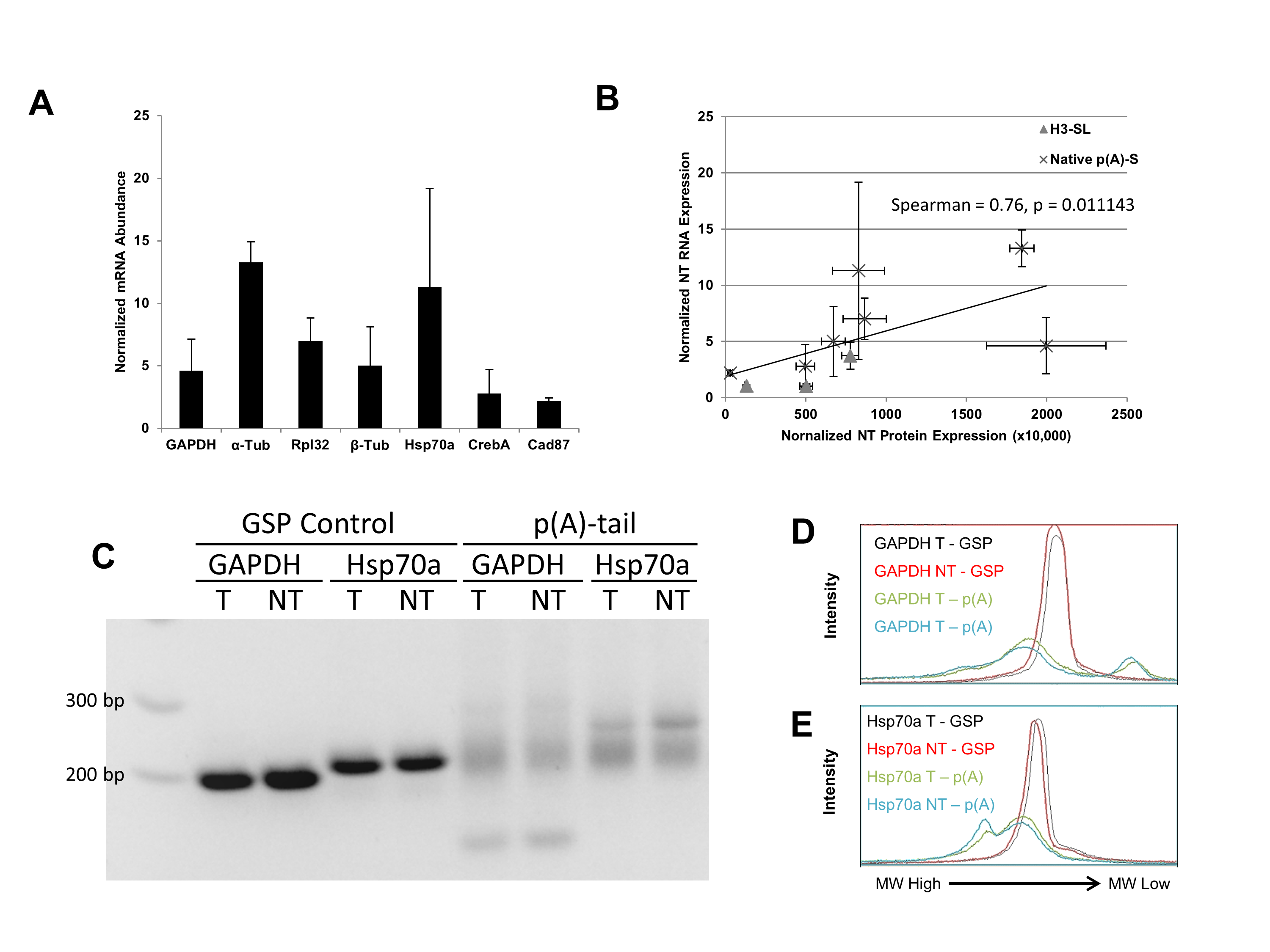


**Figure S3 | RNA abundance correlates with non-targeted reporter protein expression**

**A** Normalized mRNA abundances for each 3’UTR reporter measured in Figure 1 of the man text. **B** Correlation between mRNA abundances and NT reporter luciferase activity. All data are depicted as mean ± SD. **C** Poly-A tail length analysis for GAPDH and Hsp70a 3’UTR reporters. The p(A)-tail length was assayed using the GI-tailing approach. GSP control refers to PCR amplified using a GSP that binds at the end of the 3’UTR and a forward primer that binds at the end of the bantam target/non-target sites. For p(A)-tail PCR a p(C) primer was used. Panels **D** and **E** show the band intensity along the y-axis of the gel for each lane.

**
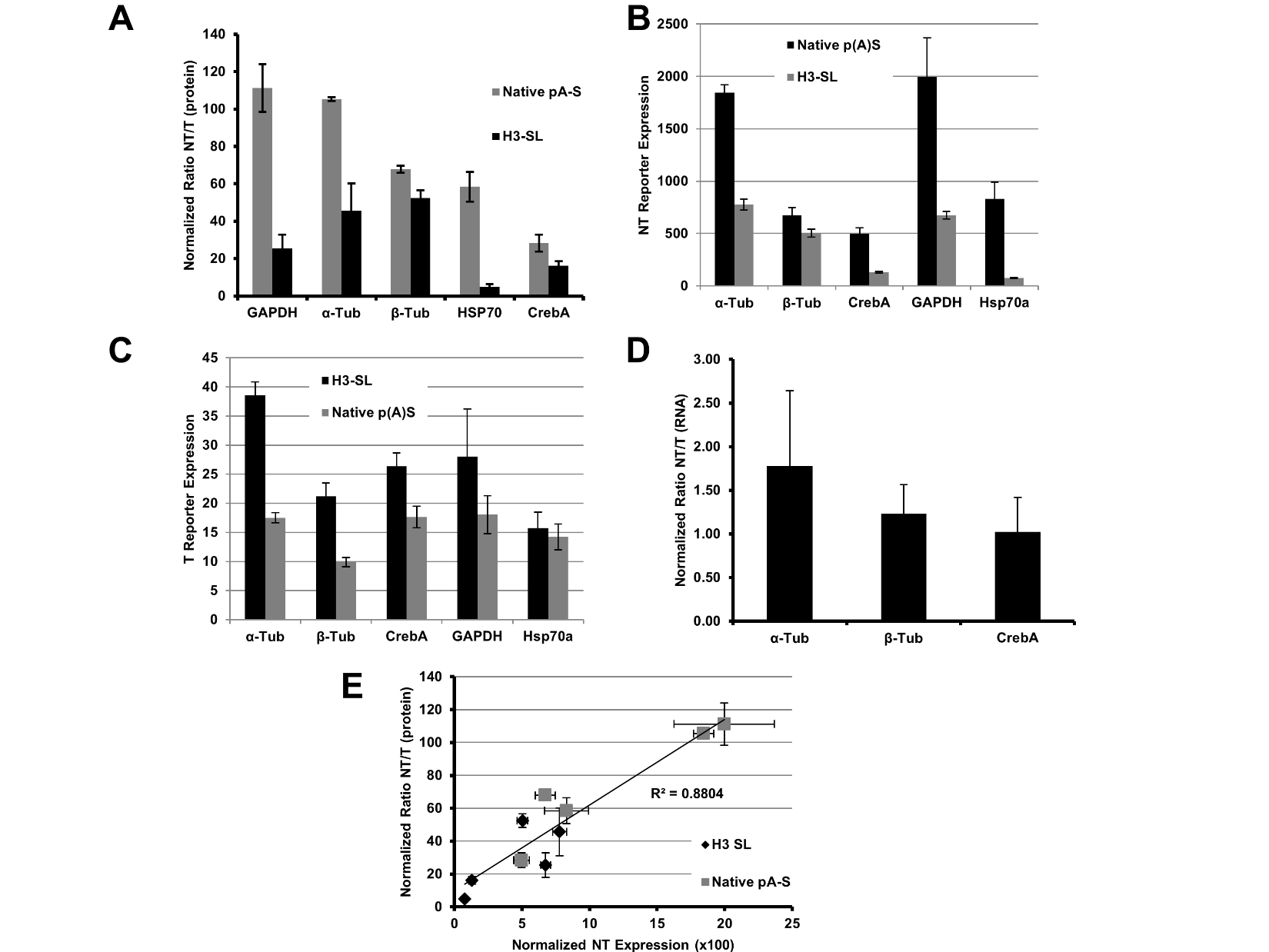
**

**Figure S4 | Reporter transcripts terminated by the histone H3 stem loop show correlation between repression and non-targeted reporter expression**

**A** The 3’UTR reporters from Figure 1 were cloned with the histone H3 stem loop (H3-SL) which results in non-poly(A)-tail and PABP driven translation through stem-loop binding protein (SLBP). Dual-luciferase assay was used to determine repression of each reporter. Normalization was carried out using firefly luciferase activity. **B** Expression levels of the NT reporters with poly(A)-tail and H3-SL. **C** Expression levels of the T reporters with poly(A)-tail and H3-SL. **D** Reporters terminated by the H3-SL show minimal miRNA-mediated mRNA degradation as measured by the ratio of NT/T *Renilla* mRNA by qRT-PCR. **E** Reporters with poly(A)-tail or the H3-SL show correlation between translatability (NT expression) and repression (normalized ratio of NT/T for luciferase activity). All data are depicted as mean ± SD.

**
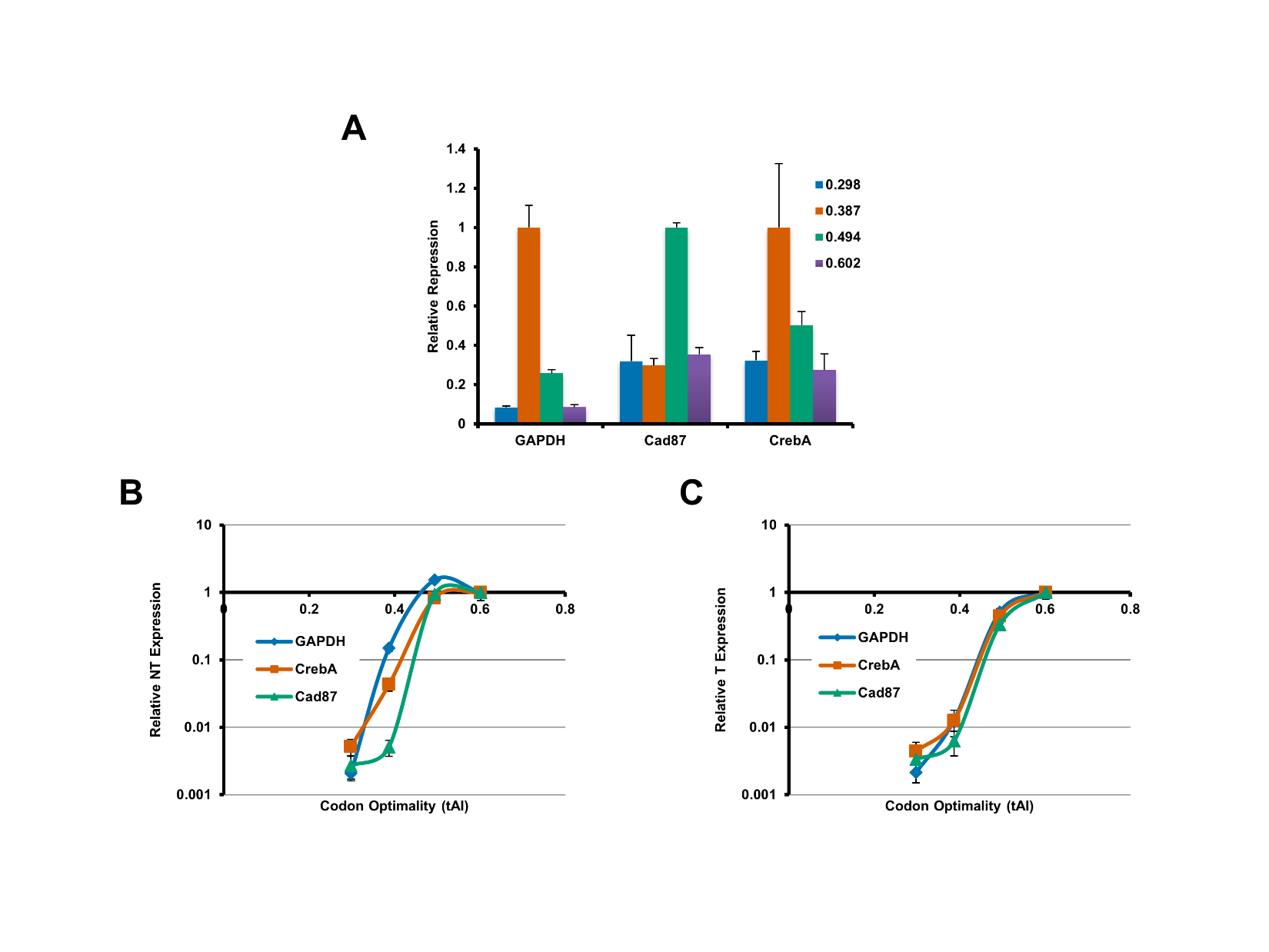
**

**Figure S5 | Reporters with moderate codon optimality show robust repression**

**A** Normalized ratio NT/T for luciferase activity from Figure 3B is shown as relative repression, with all values for each 3’UTR set relative to the maximal repression observed for each 3’UTR reporter. **B** The NT reporter expression of all 3’UTR reporters is shown. NT reporter *Renilla* luciferase activity was normalized to firefly luciferase activity and set relative to expression of the 0.602 tAI reporter. **C** The T reporter expression of all 3’UTR reporters is shown. T reporter *Renilla* luciferase activity was normalized to firefly activity and set relative to expression of the 0.602 tAI reporter. All data are depicted as mean ± SD.

**
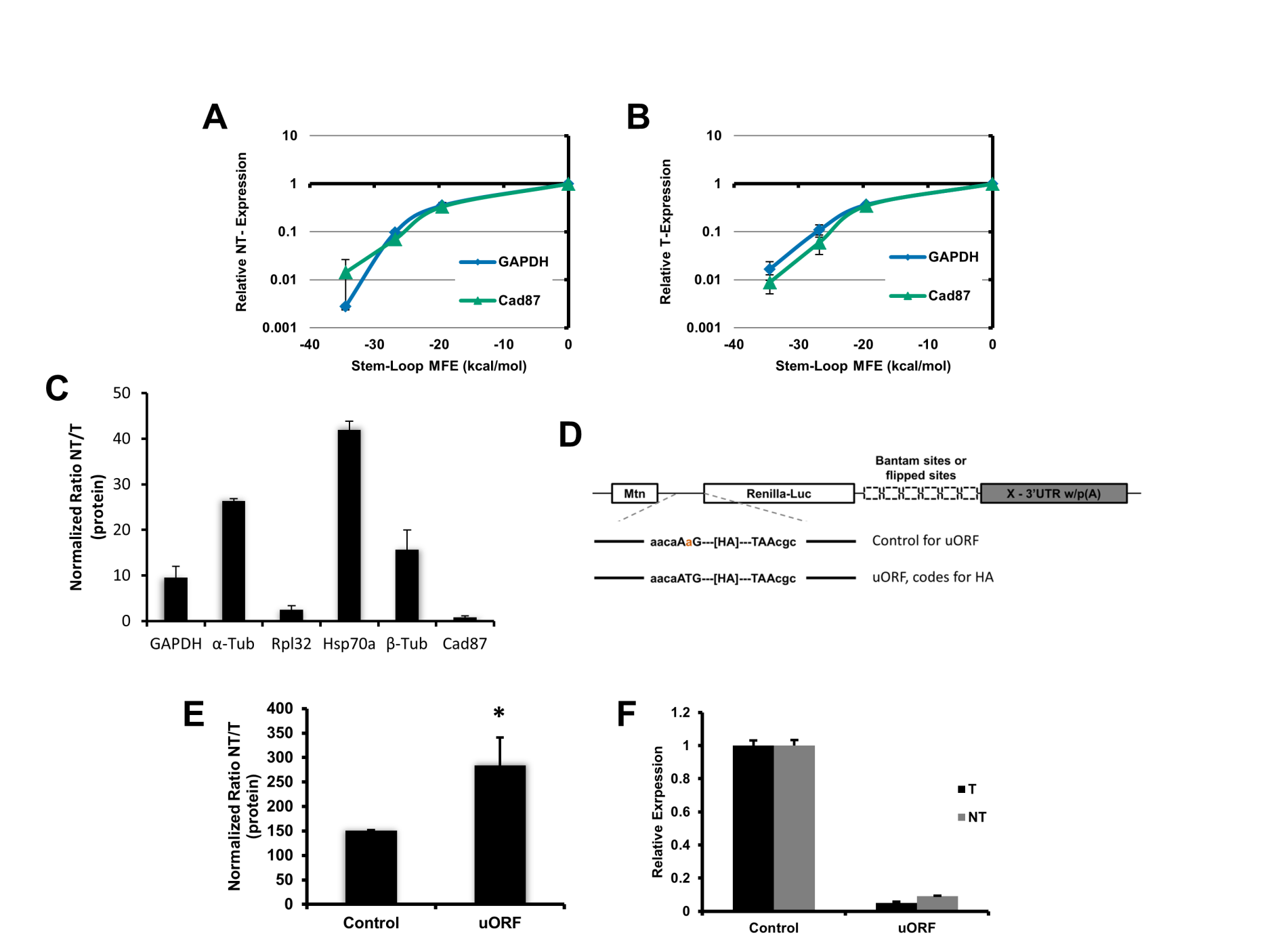
**

**Figure S6 | 5’UTR structure and uORF influence non-targeted and targeted reporters differentially**

**A** The NT reporter expression of both 3’UTR reporters is shown. NT reporter *Renilla* luciferase activity was normalized to firefly activity and set relative to expression of the SL control reporter. **B** The T reporter expression of both 3’UTR reporters is shown. T reporter *Renilla* luciferase activity was normalized to firefly luciferase activity and set relative to expression of the SL control reporter. **C** The cognate 5’UTR of each 3’UTR reporter used in Figure 1A was cloned in place of the 5’UTR in the vector pMT-DEST48. The repression of each reporter is shown. **D** Schematic describing the 5’UTR inserts used in panels **E** and **F. E** Insertion of an uORF in the 5’UTR of the GAPDH 3’UTR reporter increased repression. **F** The expression of the NT and T reporter for GAPDH was reduced upon insertion of the uORF. The expression of the T reporter was reduced more than the NT reporter. All data are depicted as mean ± SD.

**
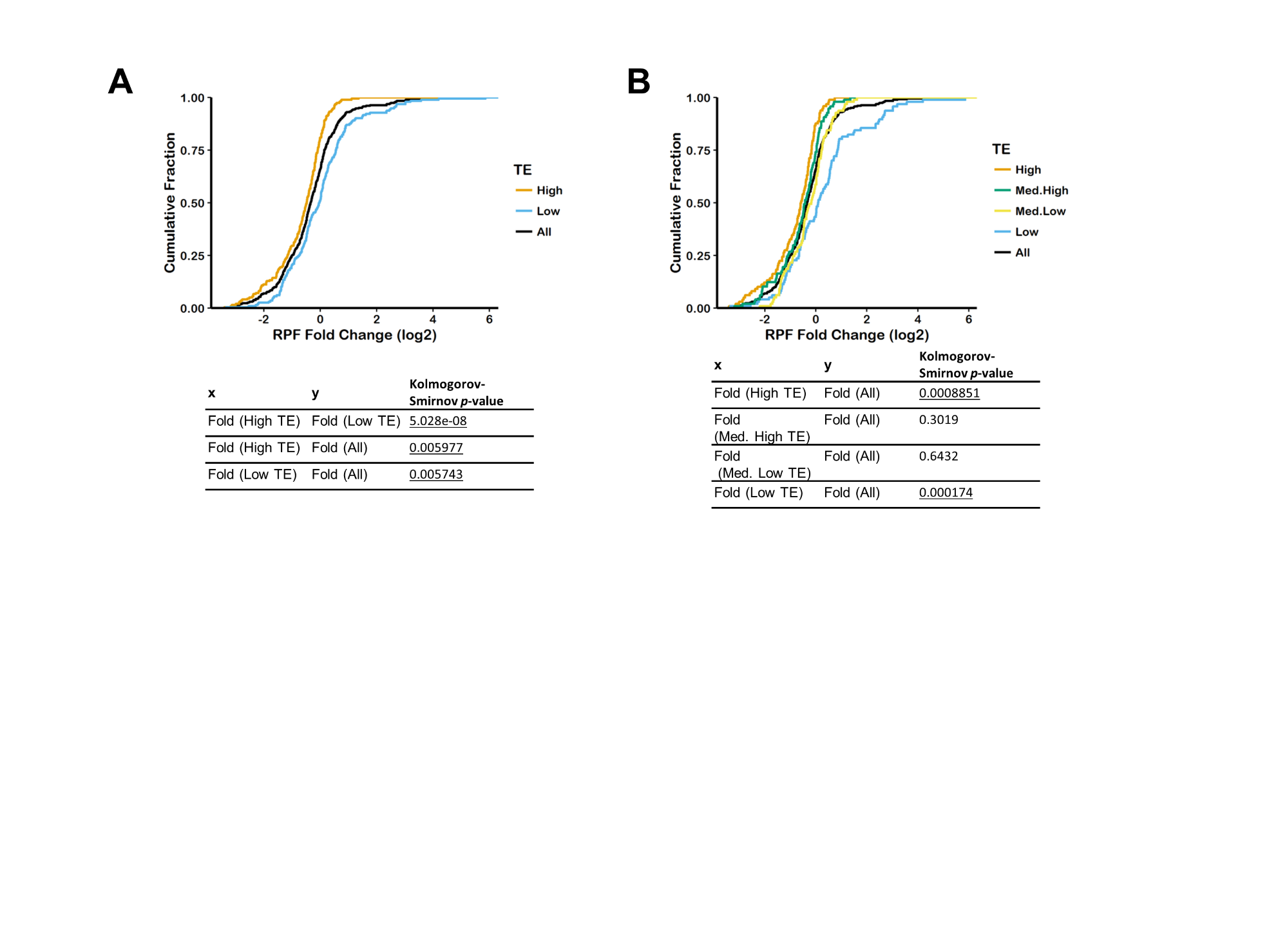
**

**Figure S7 | Statistical analysis of the interaction between TE and translational repression**

Cumulative distributions of fold change of RPFs, **A** and **B** for all miR-155 predicted targets (http://targetscan.org/) in data from Guo et al., 2010. Fold change is calculated as the log_2_ normalized RPF reads for miR-155 transfected divided by mock transfected. TE for each transcript in the absence of miR-155 (mock transfection) was calculated by normalized RPF divided by normalized RNAseq reads. All miR-155 targets are binned by TE, above or below the median (“High” or “Low”), **A**. or by TE quartiles, **B**. The tables below each plot describe the p-value for Kolmogorov-Smirnov tests of each population.


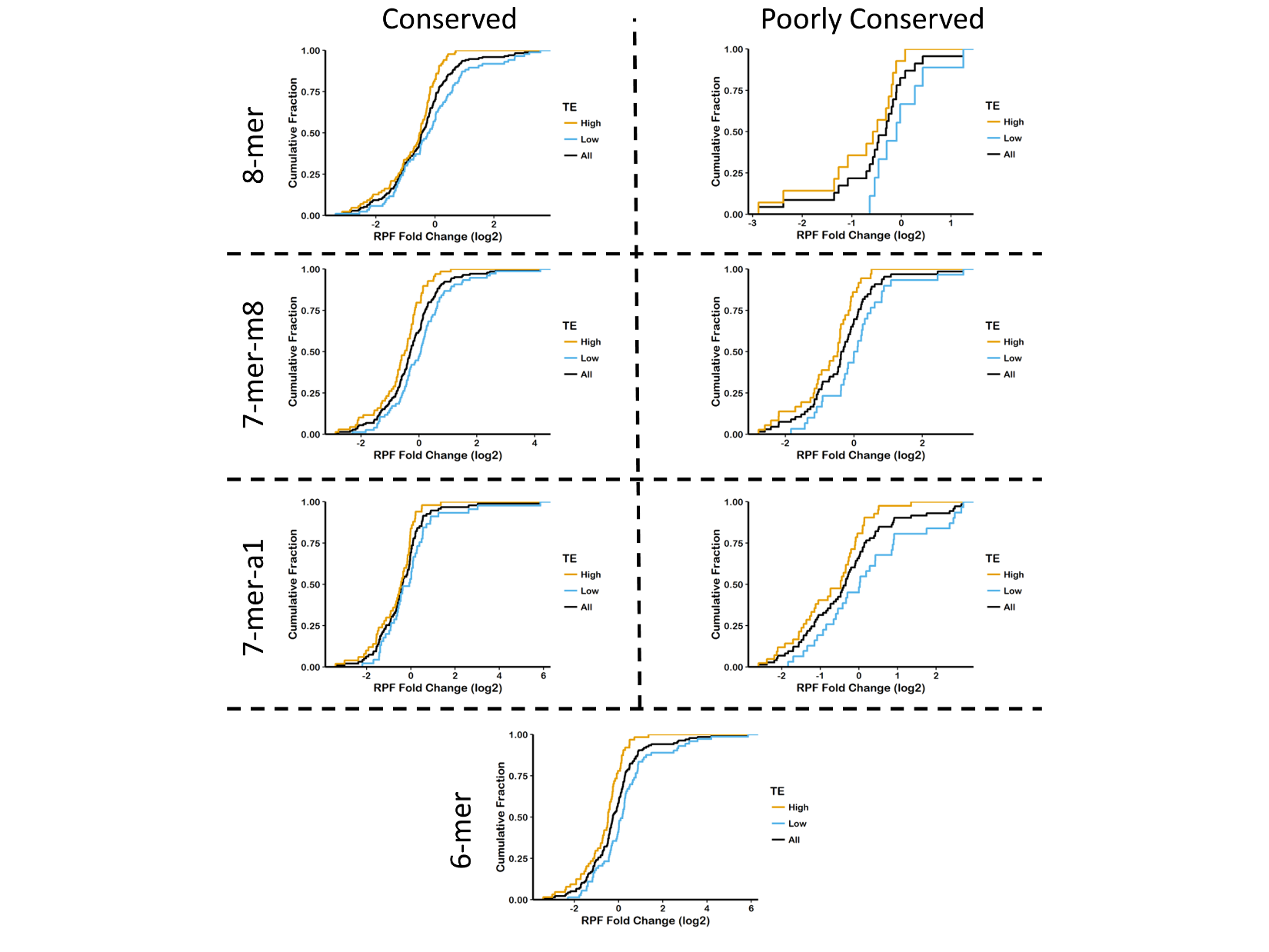


**Figure S8 | TE influences the magnitude of repression by miR-155 independent of the seed pairing**

Cumulative distributions of fold change of RPFs, for miR-155 predicted targets containing a conserved or poorly conserved 8-mer, 7-mer-a1, 7-mer-m8, or 6-mer seed pairing with miR-155 (http://targetscan.org/) in data from Guo et al., 2010. Fold change is calculated as the log_2_ normalized RPF reads for miR-155 transfected divided by mock transfected. TE for each transcript in the absence of miR-155 (mock transfection) was calculated by normalized RPF divided by normalized RNAseq reads. All miR-155 targets are binned by TE, above or below the median (“High” or “Low”).


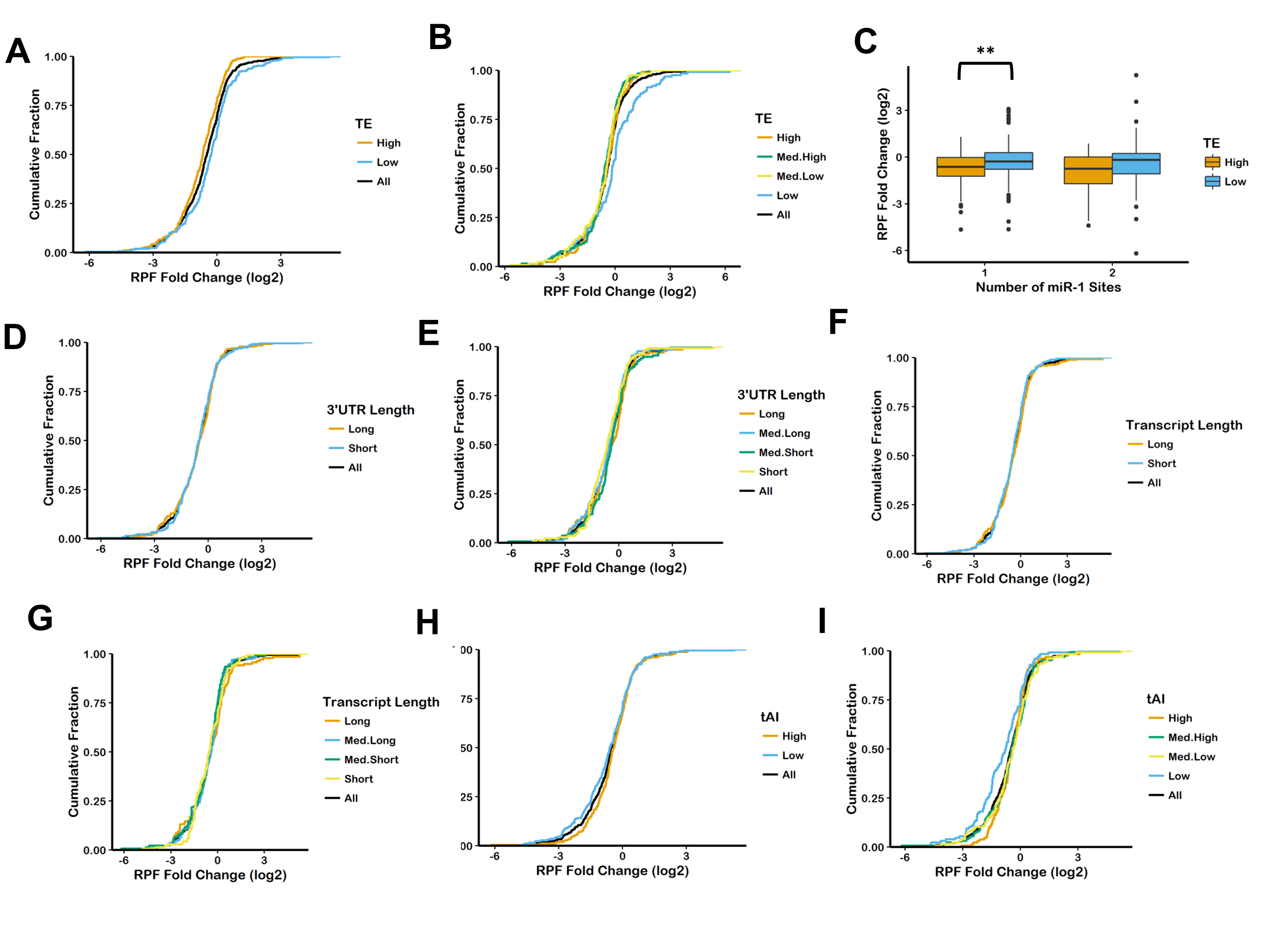


**Figure S9 | TE influences the magnitude of repression by miR-1**

Cumulative distributions of fold change of RPF or RNAseq for all miR-1 predicted targets (<http://targetscan.org/>) in data from Guo et al., 2010. Fold change is calculated as the log_2_ normalized RPF or RNAseq reads for miR-1 transfected divided by mock transfected. TE for each transcript in the absence of miR-1 (mock transfection) was calculated by normalized RPF divided by normalized RNAseq reads. All miR-1 targets are binned by TE, above or below the median (“High” or “Low”), **A** or by TE quartiles, **B**. **C**, miR-1 targets are binned by the number of conserved and poorly conserved binding sites for miR-1 as well as TE. ** *p*<0.01 by Kolmogorov–Smirnov test. **D** miR-1 targets are binned by 3’UTR length, above the median “Long”, or below the median “Short”. **E** miR-1 targets are binned by 3’UTR length into quartiles, “Long”, “Med.Long”, “Med.Short”, and “Short”. **F** All miR-1 targets are binned by transcript length, above the median “Long”, or below the median “Short”. **G** miR-1 targets are binned by transcript length into quartiles, “Long”, “Med.Long”, “Med.Short”, and “Short”. **H** miR-1 targets are binned by tAI, above the median “High”, or below the median “Low”. **I** miR-1 targets are binned by tAI into quartiles, “High”, “Med.High”, “Med.Low”, and “Low”.

**
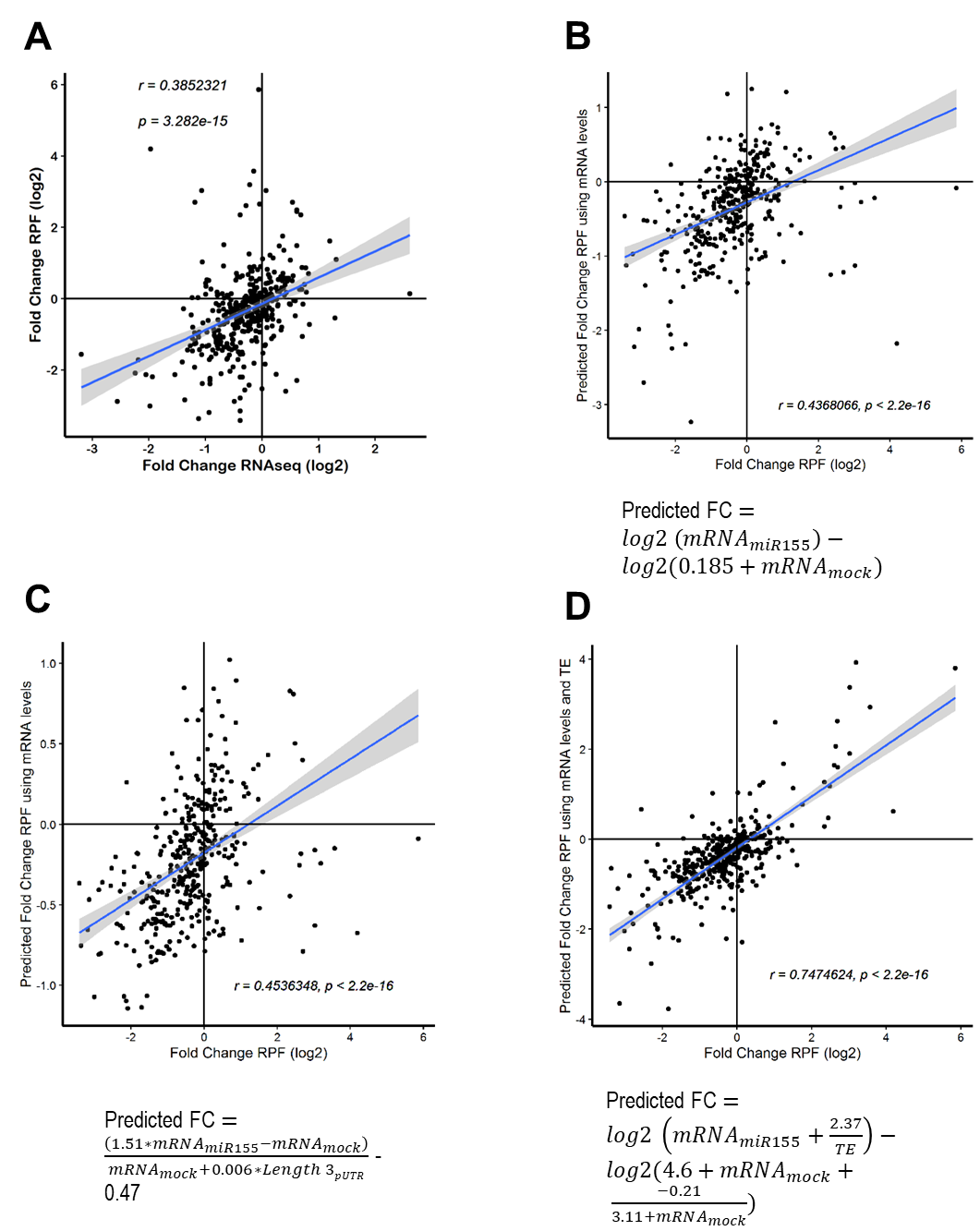
**

**Figure S10 | TE along with mRNA abundance can predict fold change of RPF**

Plots showing correlation between measured FC and predicted based on the different equations derived from the data for miR155 repressed sample. For the prediction we have chosen not the best equation but the one that was among the best and among the simplest. These typically were not scoring worse than 0.05 in R-goodness-of-fit units compared to the complex versions. **A** Correlation between Fold Change RPF and Fold Change of mRNA from corresponding RNAseq experiments. **B** Prediction of Fold Change RPF using only mRNA levels and the corresponding equation derived by Eureqa. **C** Best prediction of Fold Change RPF using more variables than mRNA levels and the corresponding equation derived by Eureqa. Only length of 3’ UTR was selected as the variable improving the model. However, the selected equation does not make sense mathematically, as FC is a logarithm. **D** Prediction of FC using mRNA levels and TE and the corresponding equation derived by Eureqa.


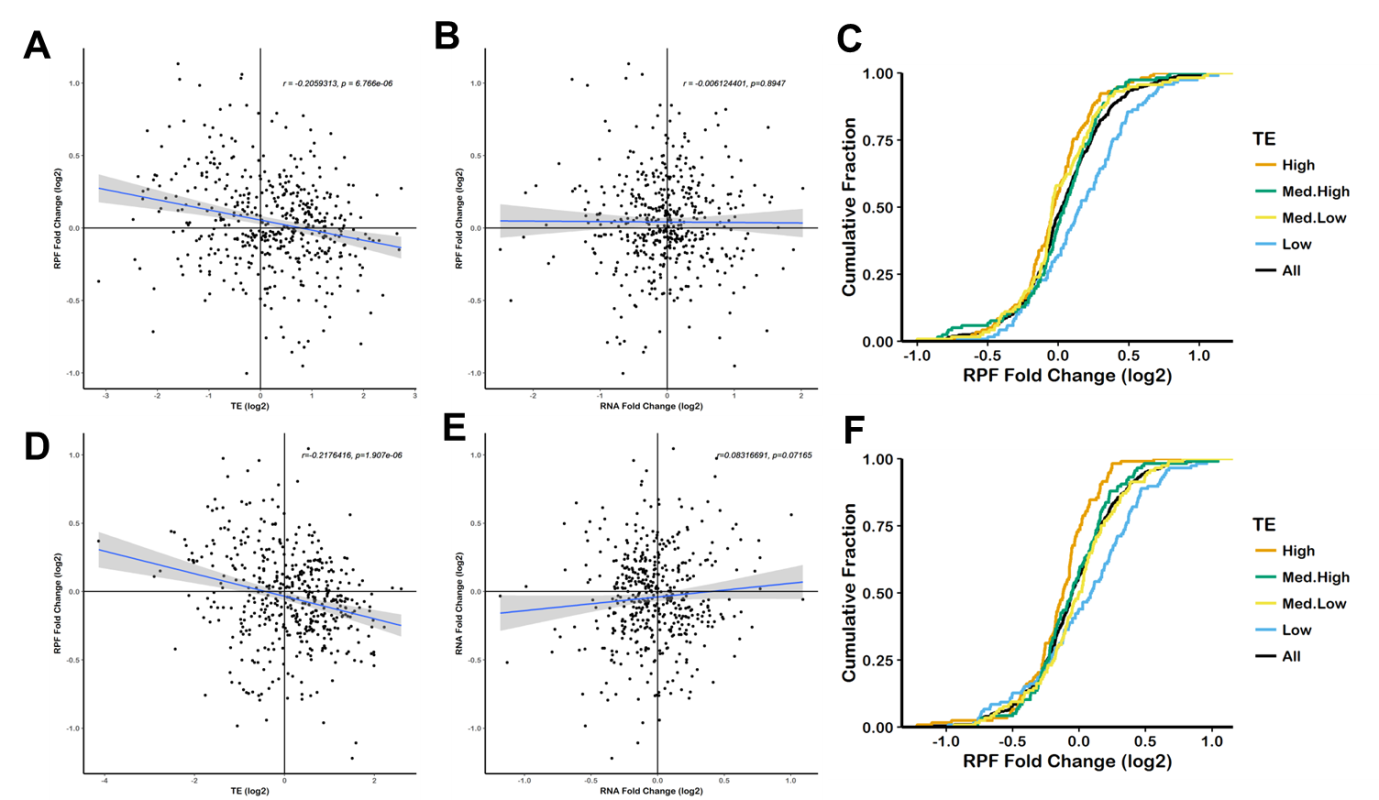


**Figure S11 | Correlation between TE and RPF fold change for miR-155 targets in LPS activated B-cells**

Correspondence between RPF fold change and TE, **A** and **D**, or RNA fold change and TE, **B** and **E**, at 2 hr, **A** and **D**, or 4 hr, **B** and **E**, post activation of B-cells with LPS in data from Eichorn et al., 2014. Fold change is calculated as the log_2_ normalized RPF or RNAseq reads for WT B-cells divided by miR-155 knockout B-cells. TE for each transcript in the absence of miR-155 (knockout) was calculated by normalized RPF divided by normalized RNAseq reads. Cumulative distributions of fold change of RPF, **C** and **F**, for all miR-155 predicted targets in B-cells at 2 hr post activation, **C**, or 4 hr post activation, **F**. All miR-155 targets are binned by TE quartiles.


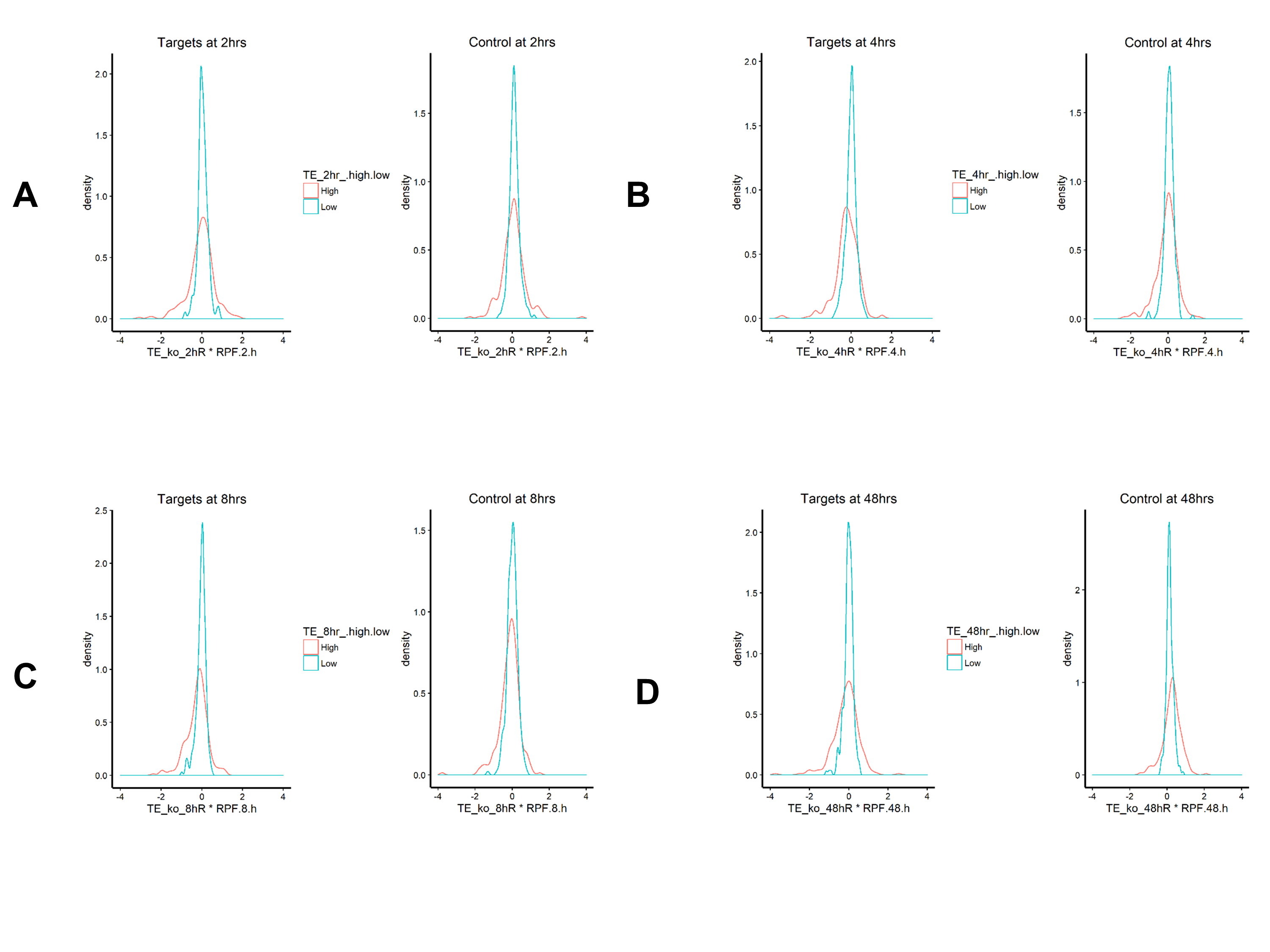


**Figure S12 | Comparison of distributions of TE multiplied by RPF Fold Change across groups and time points.** Across time points TE*RPF FC gradually shifts toward left for targets and stays the same or shifts towards right for non-targeted genes. Statistics calculated using Kolmogorov-Smirnov test. **A**. 2hrs, p-value that TE*RPF_FC is smaller for targets than for control: 0.002756 **B**. 4hrs, p-value that TE*RPF_FC is smaller for targets than for control: 8.037e-05 **C**. 8 hrs, p-value that TE*RPF_FC is smaller for targets than for control: 0.003012 **D**. 48 hrs, p-value that TE*RPF_FC is smaller for targets than for control: < 2.2e-16.


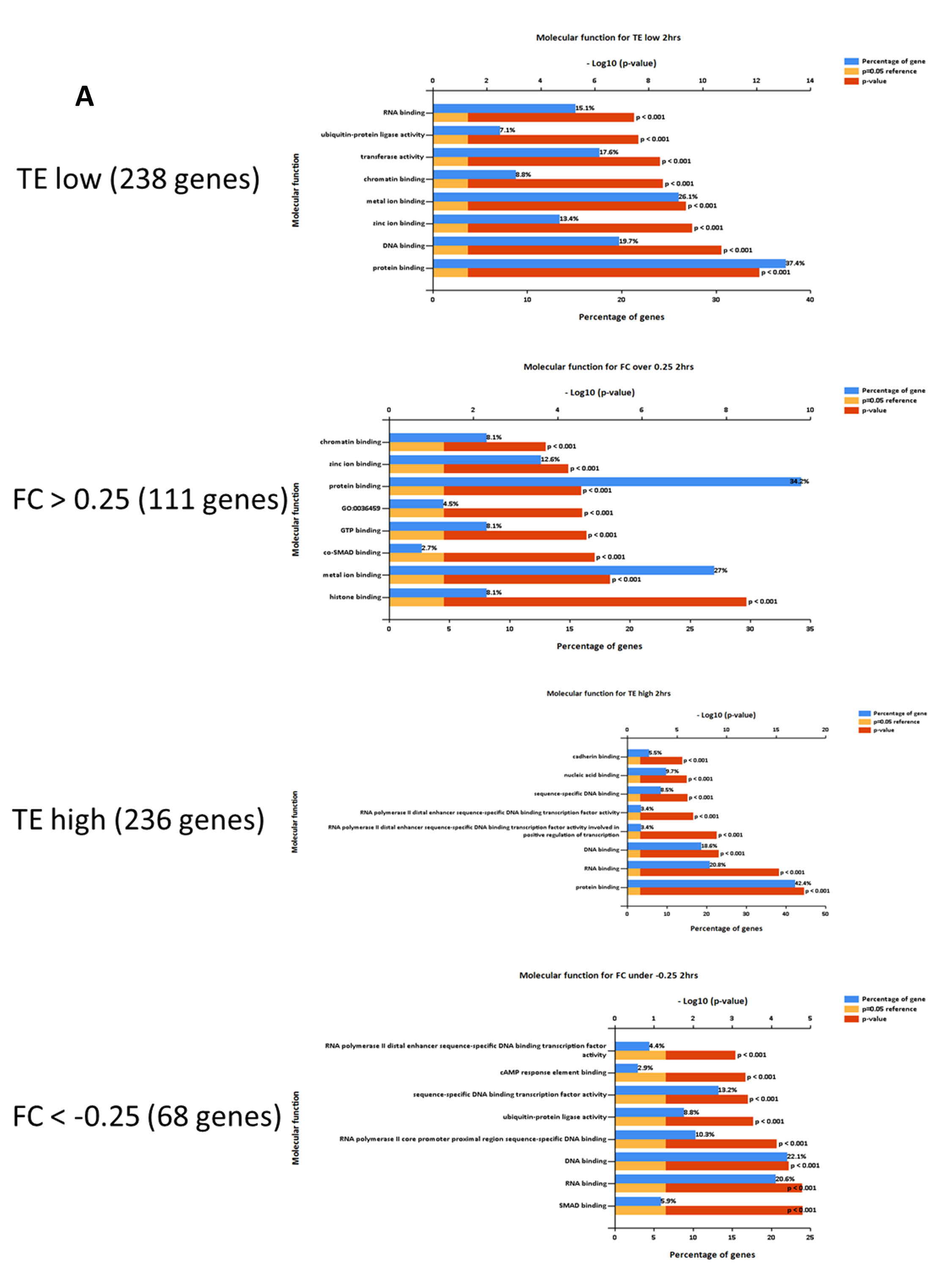


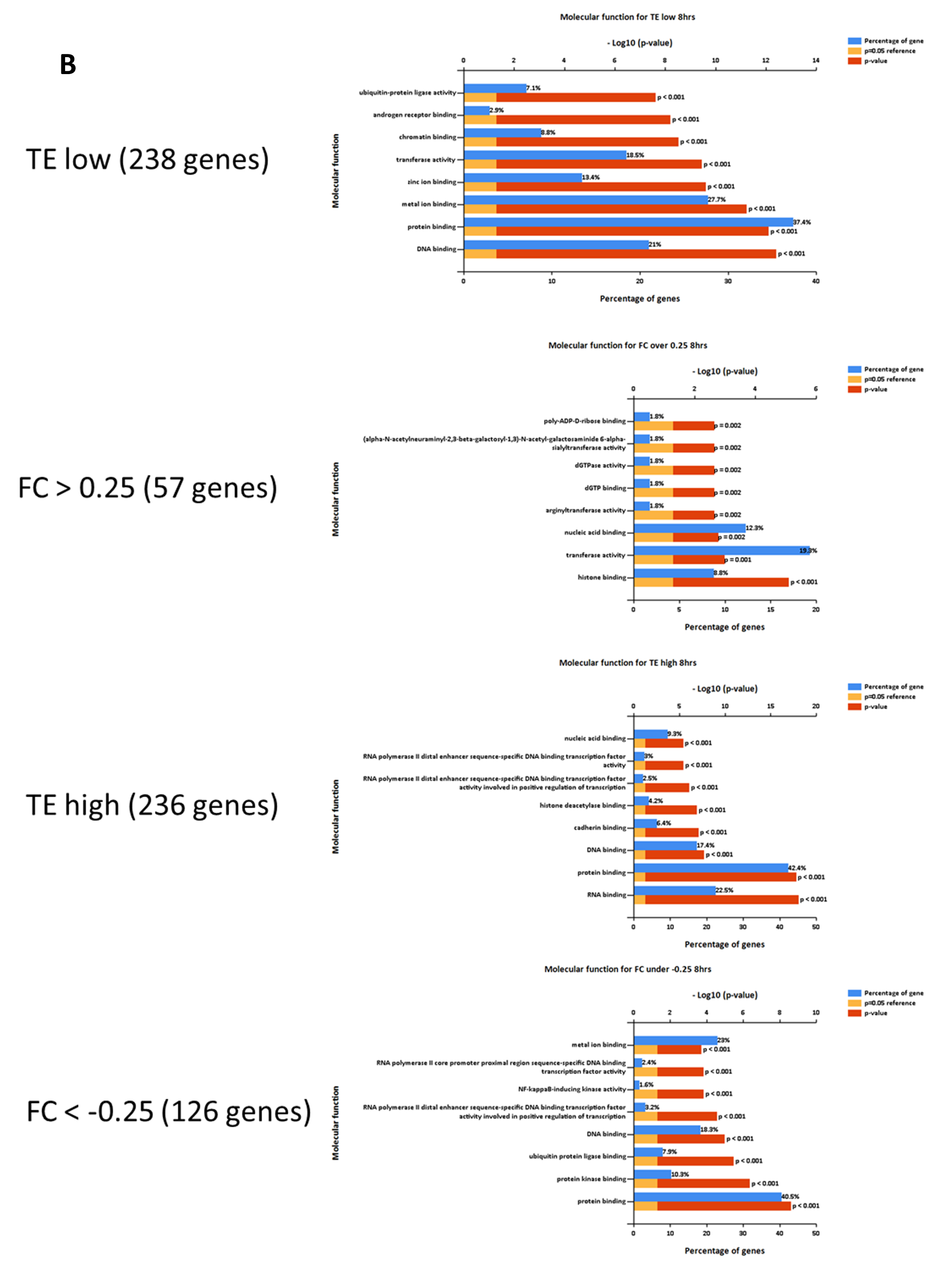


# Figure S13 | Gene ontology analysis for 4 groups: TE high, TE low and RPF FC below -0.25 and RPF FC > 0.25. Eight most statistically significant terms are provided for each group. Timepoints: A 2hrs, B 8hrs. Highly repressed genes (FC values below -0.25 or TE high) have a similar functional profiles. Due to small sets used in case of fold-change-selected groups, uncorrected p-values are reported in all cases.


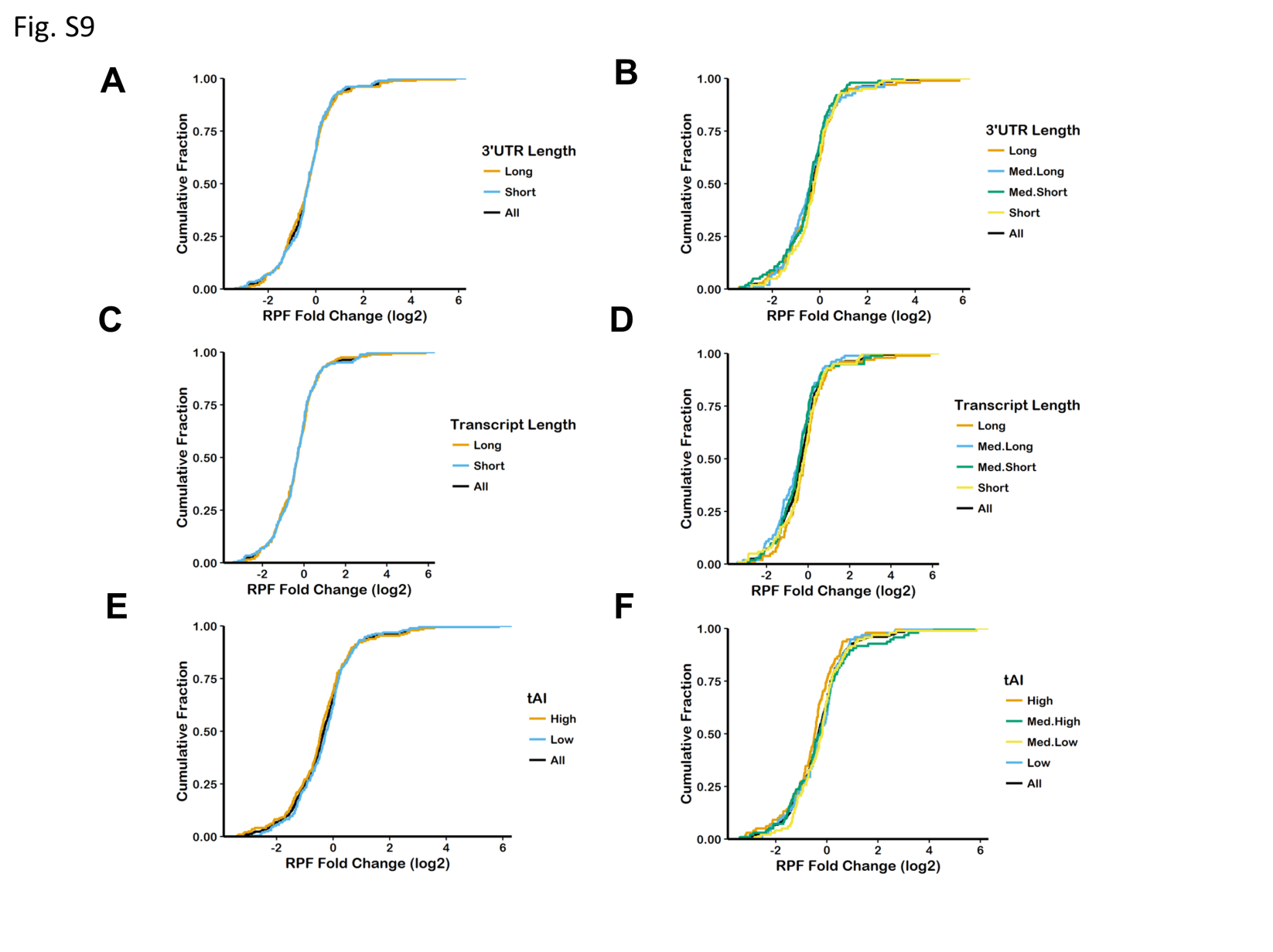


**Figure S14 | 3’UTR length, transcript length and tAI do not influence fold change of RPF**

Cumulative distributions of fold change of RPF for all miR-155 predicted targets (targetscan.org) in data from Guo et al., 2010. Fold change is calculated as the log_2_ normalized RPF for miR-155 transfected divided by mock transfected. **A** We binned all miR-155 targets by 3’UTR length, above the median “Long”, or below the median “Short”. **B** We binned all miR-155 targets by 3’UTR length into quartiles, “Long”, “Med.Long”, “Med.Short”, and “Short”. **C** We binned all miR-155 targets by transcript length, above the median “Long”, or below the median “Short”. **D** We binned all miR-155 targets by transcript length into quartiles, “Long”, “Med.Long”, “Med.Short”, and “Short”. **E** We binned all miR-155 targets by tAI, above the median “High”, or below the median “Low”. **F** We binned all miR-155 targets by tAI into quartiles, “High”, “Med.High”, “Med.Low”, and “Low”.


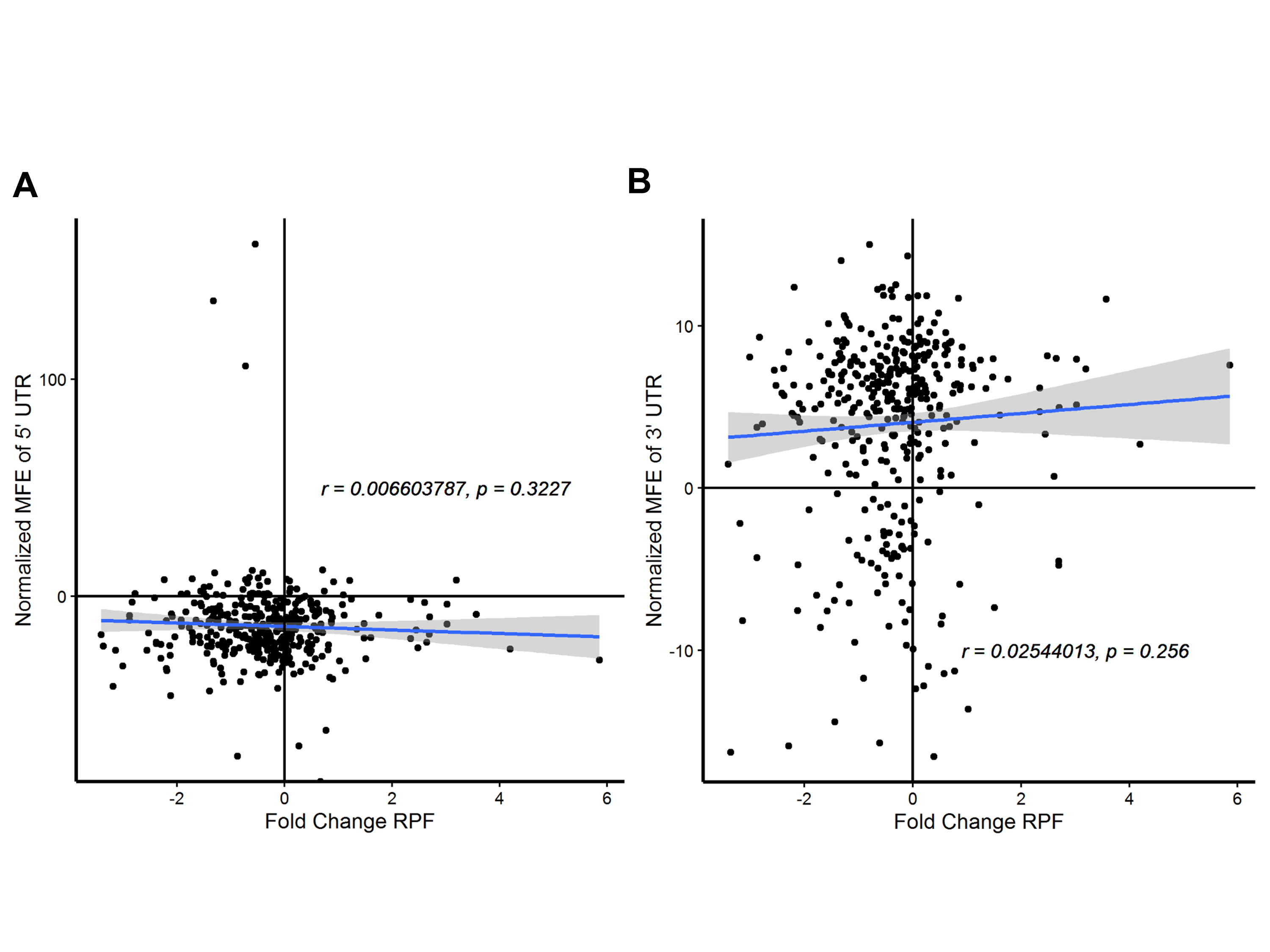


**Figure S15 | 5’UTR and 3’UTR structure do not correlate with fold change of RPF**

Lack of correlation between Fold Change RPF and normalized MFEs of 5’ UTR, **A**, and 3’ UTR, **B**, of miR-155 targeted genes. Normalization of MFE was done according to Trotta method (Trotta, 2014) which removes all dependence of sequence length from the final MFE values.

**
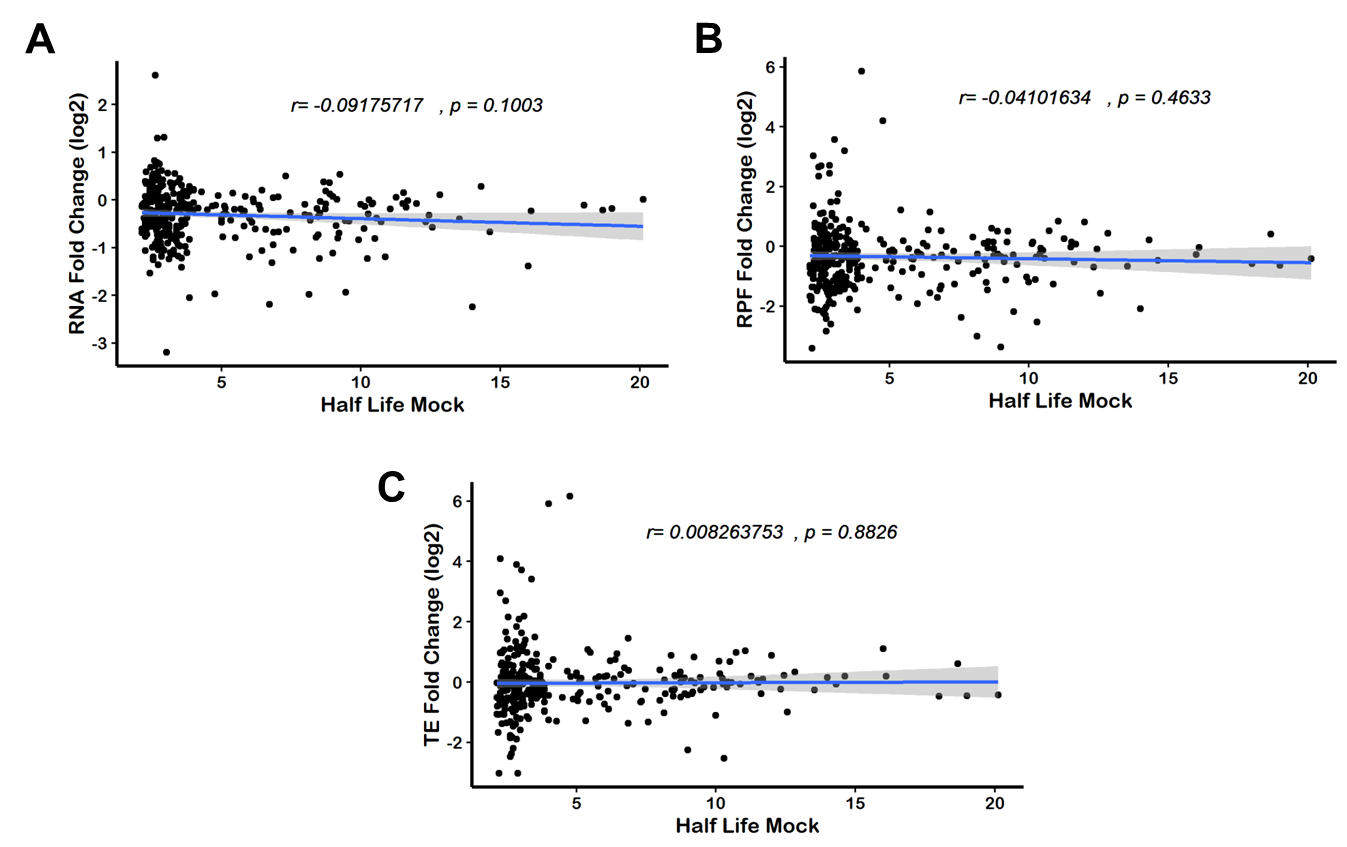
**

**Figure S16 | mRNA half-life does not correlate with Fold Change of RPF, RNA or TE**

Lack of correlation between miR-155 induced Fold Change of RNA, **A**, RPF, **B**, and TE, **C**, and mRNA half-life in untransfected HeLa, Tani et al., 2012.

**Table S1.**

**Primers and oligonucleotides**

| **Primer/Oligo Name** | **Sequence (5’->3’)** |
| --- | --- |
| *Renilla* luc. forward | CACCATGGGCACTTCGAAAGTTTATG |
| HID/FLP reverse | GTATCTTATCATGTCTGCTCGAAGCG |
| SV40 p(A)s mut. forward | CCATTATAAGCTGCCAAGTTAACAACAACAATTGC |
| SV40 p(A)s mut. reverse | GCAATTGTTGTTGTTAACTTGGCAGCTTATAATGG |
| GAPDH overlap forward | GCTTCGAGCAGACATGATAAGATACACTAGCCAAAACTATCGTACAAACC |
| GAPDH reverse | CTTCATTCGATGCACAAGTTTTATTTTTC |
| α-tubulin overlap forward | CGCTTCGAGCAGACATGATAAGATACGCGTCACGCCACTTCAACG |
| α-tubulin reverse | CTTATTTCTGACAACACTGAATCTG |
| β-tubulin overlap forward | CGCTTCGAGCAGACATGATAAGATACATTCGAATCGGAAATCAATCGAATTC |
| β-tubulin reverse | AGACTTGTGAACAAAATTGGATCCG |
| Rpl32 reverse w/overlap | CATTTTTTAACTAAAAGTCCGGTATATTAACGTTTACAAATGTGTATTCCGACCACGTTACAAGAACTCTCAAGAATCTTAAGCGTATCTTATCATGTCTGCTCG |
| Hsp70a overlap forward | GCTTCGAGCAGACATGATAAGATACGGCCAAAGAGTCTAATTTTTGTTC |
| Hsp70a reverse | AAATTCAATAAATAATTTATTTTTTCTATAAGC |
| CrebA overlap forward | CGCTTCGAGCAGACATGATAAGATACACAACCGGATTCACATGGAC |
| CrebA reverse | CAGATTCCTGCTGTTTGTATGG |
| Cad87 overlap forward | GCTTCGAGCAGACATGATAAGATACGGGTCGATGGGAACTGTTG |
| Cad87 reverse | GTATGTGTATATGTTTAATGTAAATGCAAAC |
| H3-SL oligo 1 | AATAATCGGTCCTTTTCAGGACCACAAACCAGATTCAATGAGATAAAATTTTCTGTT |
| H3-SL oligo 2 | AACAGAAAATTTTATCTCATTGAATCTGGTTTGTGGTCCTGAAAAGGACCGATTATT |
| GAPDH –p(A)s reverse | CAACAACAATAAATATGTAGCTTTGC |
| α-tubulin –p(A)s reverse | CTTGTGTACACAACTTATCGCC |
| β-tubulin –p(A)s reverse | GATTACGTTGTTAAGAGAACAAATC |
| Hsp70a –p(A)s reverse | CTATAAGCAATAACATTTTTGCTAAATTAAG |
| CrebA –p(A)s reverse | ACAATATTATTATTTAGCTTCTCTTTAG |
| Control 5’UTR insert oligo 1 | CAACAACAACAACAACAACAACCAACAACAACAACAACAACAACAAGC |
| Control 5’UTR insert oligo 2 | TTGTTGTTGTTGTTGTTGTTGTTGGTTGTTGTTGTTGTTGTTGTTGGC |
| 9-SL 5’UTR insert oligo 1 | CAACAACAACACGGCCCAAGCTTGGGCCGTGCAACAACAACAACAAGC |
| 9-SL 5’UTR insert oligo 2 | TTGTTGTTGTTGTTGCACGGCCCAAGCTTGGGCCGTGTTGTTGTTGGC |
| 12-SL 5’UTR insert oligo 1 | CAACAACACCACGGCCCAAGCTTGGGCCGTGGTGCAACAACAACAAGC |
| 12-SL 5’UTR insert oligo 2 | TTGTTGTTGTTGCACCACGGCCCAAGCTTGGGCCGTGGTGTTGTTGGC |
| 15-SL 5’UTR insert oligo 1 | CAACTCCACCACGGCCCAAGCTTGGGCCGTGGTGGAGCAACAACAAGC |
| 15-SL 5’UTR insert oligo 2 | TTGTTGTTGCTCCACCACGGCCCAAGCTTGGGCCGTGGTGGAGTTGGC |
| uORF Control insert oligo 1 | AACAAAGGCGTATCCGTACGACGTGCCGGATTACGCGTAACGC |
| uORF Control insert oligo 2 | GTTACGCGTAATCCGGCACGTCGTACGGATACGCCTTTGTTGC |
| HA uORF insert oligo 1 | AACAATGGCGTATCCGTACGACGTGCCGGATTACGCGTAACGC |
| HA uORF insert oligo 2 | GTTACGCGTAATCCGGCACGTCGTACGGATACGCCATTGTTGC |
| 3’RACE RT | GGCGCTAGCTGTTACTGGGCCACCACGCGTCGACTAGTACTTTTTTTTTTTTTTTTTT |
| 3’RACE External Amp | GGCGCTAGCTGTTACTGGGC |
| 3’RACE Amplification | CACCACGCGTCGACTAGTAC |
| Renilla-tail Forward | GGGAAAATATATCAAATCGTTCGTTG |
| *Renilla* luc. qRT forward | GGTATGGGCAAATCAGGC |
| *Renilla* luc. qRT reverse | GCACCCCAATCATGGCCG |
| *Renilla* 0.602 luc. qRT F | CCTACGAGCACCAGGACAAG |
| *Renilla* 0.602 luc. qRT R | CGATGTCCTCCTCGATGTCG |
| *Renilla* 0.494 luc. qRT F | GCTTTTGAACCTGCCCAAGAAG |
| *Renilla* 0.494 luc. qRT R | CCACGACTCAATCACGTCCAC |
| *Renilla* 0.298 luc. qRT forward | TGCAGCATCATCTTATCTATGGAG |
| *Renilla* 0.298 luc. qRT reverse | TCCCAGATTTACCTGACTTCCC |
| Firefly luc. qRT forward | CCAGGGATTTCAGTCGATGT |
| Firefly luc. qRT reverse | AATCTCACGCAGGCAGTTCT |
| GAPDH 5’UTR F | CCAATGtGCATCAGTTGTGGCCATTCTCCTAATTTGCGAAAAAAGC |
| GAPDH 5’UTR R | GTGCCCATGGGGCTGAGTTCCTGCTGTCTTTTC |
| α-tubulin 5’UTR F | CCAATGtGCATCAGTTGTGGTCATATTCGTTTTACGTTTGTCAAGC |
| α-tubulin 5’UTR R | GTGCCCATGGATTGAGTTTTTATTGGAAGTGTTTCAC |
| β-tubulin 5’UTR F | CCAATGtGCATCAGTTGTGGAATGCACTAATTTTTCCAAGTGTG |
| β-tubulin 5’UTR R | GTGCCCATGGTTTGTATTTGTTTTAGGCTTTTGAAC |
| Cad87 5’UTR F | CCAATGtGCATCAGTTGTGGTATGTTTTCAACAACTTCTCTCTGC |
| Cad87 5’UTR R | GTGCCCATGGTTTAGGGTCTTTAATACTGATTATCACTC |
| Hsp70a 5’UTR F | CCAATGtGCATCAGTTGTGGTCAATTCTATTCAAACAAGTAAAGTGAAC |
| Hsp70a 5’UTR R | GTGCCCATGGTGTGTGTGAGTTCTTCTTCCTCG |
| Rpl32 5’UTR F | CCAATGtGCATCAGTTGTGGTTTCTTTTCGCTTCTGGTTTCCGGCAAGCTTCAAGC |
| Rpl32 5’UTR R | CATGGCTTGAAGCTTGCCGGAAACCAGAAGCGAAAAGAAACCACAACTGATGCACATTGG |
| Renilla overlap R | GTATCTTATCATGTCTGCTCGTTATTGTTCATTTTTGAGAAC |
| HID/FLP F | CACCCGCTTCGAGCAGACATGATAAGATAC |
| GAPDH R2 | ATTACAGTAACAGGGCGATACTTTATTC |

Table S2.

Expression of miRNAs in S2 cells

| **miRNA** | **Predicted 3'UTR Target** | **Relative Expression RNAseq^1^** | **Relate Expression qPCR** |
| --- | --- | --- | --- |
| bantam | N/A | 100.00 | 100.00 |
| miR-1 | CrebA | 0.00 | ND |
| miR-7 | Cad87 | 2.11 | 1.12 |
| miR-8 | Hsp70a | 23.16 | 60.87 |
| miR-10-3p | Hsp70a, Cad87 | 0.00 | ND |
| miR-14 | Hsp70a | 335.42 | 752.97 |
| miR-252 | Cad87 | 75.32 | 9.76 |
| miR-263a | Cad87 | 0.59 | 3.52 |
| miR-274 | GAPDH | 0.08 | 0.13 |
| miR-277 | Cad87 | 18.68 | 20.05 |
| miR-283 | GAPDH | 0.42 | 9.11 |
| miR-304 | Rpl32 | 0.08 | 1.30 |
| miR-316 | Cad87 | 0.00 | ND |
| mIR-956 | Cad87 | 0.00 | ND |
| mIR-964 | Cad87 | 0.00 | ND |
| miR-965 | α-tubulin | 1.18 | 0.00 |
| miR-981 | Cad87 | 0.00 | ND |
| miR-987 | Cad87 | 0.00 | ND |
| miR-999 | α-tubulin, CrebA, Cad87 | 0.76 | 0.52 |
| miR-1000 | Cad87 | 0.00 | ND |
| miR-1002 | Cad87 | 0.00 | ND |
| miR-1006 | Cad87, Hsp70a | ND | 0.07 |
| miR-1014 | CrebA, Hsp70a | ND | 0.05 |

ND = not determined

Table S3

Primers for miRNA qRT-PCR

| **miRNA** | **RT Primer (5’-> 3’)** | **qPCR Reverse (5’-> 3’)** | **qPCR Forward (5’-> 3’)** |
| --- | --- | --- | --- |
| Bantam | ACTTTAAGCCGAGTCAATAATCAGCTTTC | TTTGCCTTGCTGAGATCATTTTGA | TGAACTTTAAGCCGAGTCAATA |
| mir-7 ^2^ | AGCATTCGTCTCGACACAGCAACAAAATC | TGACTCTGCTGGAAGACTAGTGAT | TAGAGCATTCGTCTCGACACAG |
| miR-274 | GAAGCTCGCATTACAGTGTACCCGTTAGT | GACAGACCTCTTTGTGACCGACAC | AGCGAAGCTCGCATTACAGTGT |
| miR-283 | TACTCACAAACTGTGATCCCAGAATTACC | CTACTTAGATAAATATCAGCTGGT | AAGTACTCACAAACTGTGATCC |
| miR-304 | CACCACAAGTCAAGCCATCTCACATTTAC | TGCCACACATTAATCTCAATTTGT | AGGCACCACAAGTCAAGCCATC |
| miR-999 | TGCCTCTCTGACACGCCGAGACACAGTCT | TGAGCTAGTCTGTTAACTGTAAGA | CTTTGCCTCTCTGACACGCCGA |
| miR-965 | CGATCCGTTCAGTCTCGTAAGGGGAAAAG | AGTGAGCGATTAAGCGTATAGCTT | CCTCGATCCGTTCAGTCTCGTA |
| miR-277 | CCGAGGGCACAATCCTATTGTCGTACCAG | AAGCTTGACGTAAATGCACTATCT | GATCCGAGGGCACAATCCTATT |
| miR-252 | TCCCATTTGTATGTTCAGCTCCTGCGGCA | CGCTTTCAAGCTAAGTACTAGTGC | GACTCCCATTTGTATGTTCAGC |
| miR-263a | CCGAAGTTAAGTAGGTCGCCCGTGAATTC | GGTCGCGAGTAATGGCACTGGAAG | CAGCCGAAGTTAAGTAGGTCGC |
| miR-1014 | TGAGATGCCATGGAGGCTCTGCAAATGAA | ACGTGAACTAAAAATTCATTTTCA | CGCTGAGATGCCATGGAGGCTC |
| miR-1006 | CTCGTTCATCCCGTGGGACTATGAATAAG | AGGCTCCGGATAAATTCGATTTCT | GTGCTCGTTCATCCCGTGGGAC |
| miR-14 | TTCTGAATTGTGCAATCCATAGGAGAGAG | TACTTGGGTTTCAGTCTTTTTCTC | GTATTCTGAATTGTGCAATCCA |
| miR-8 | CTCCACCTTATCTAAGCTGACATCTTTAC | TGATCTCGCCTAATACTGTCAGGT | TCTCTCCACCTTATCTAAGCTG |

1. Ruby, J.G. et al. Evolution, biogenesis, expression, and target predictions of a substantially expanded set of Drosophila microRNAs. *Genome Res* **17**, 1850-1864 (2007).

2. Wang, X. A PCR-based platform for microRNA expression profiling studies. *RNA* **15**, 716-723 (2009).
